# Heritability of functional gradients in the human subcortico-cortical connectivity

**DOI:** 10.1038/s42003-024-06551-5

**Published:** 2024-07-12

**Authors:** Xinyu Wu, Yu Zhang, Mufan Xue, Jinlong Li, Xuesong Li, Zaixu Cui, Jia-Hong Gao, Guoyuan Yang

**Affiliations:** 1 Advanced Research Institute of Multidisciplinary Sciences, Beijing Institute of Technology, Beijing, China; 2 School of Computer Science and Technology, Beijing Institute of Technology, Beijing, China; 3 Chinese Institute for Brain Research, Beijing, China; 4 Beijing City Key Lab for Medical Physics and Engineering, Institution of Heavy Ion Physics, School of Physics, Peking University, Beijing, China; 5 Center for MRI Research, Academy for Advanced Interdisciplinary Studies, Peking University, Beijing, China; 6 McGovern Institute for Brain Research, Peking University, Beijing, China; 7 Institute of Artificial Intelligence, Hefei Comprehensive National Science Center, Hefei, China; 8 School of Medical Technology, Beijing Institute of Technology, Beijing, China

**Keywords:** Genetics of the nervous system, Neural circuits

## Abstract

The human subcortex plays a pivotal role in cognition and is widely implicated in the pathophysiology of many psychiatric disorders. However, the heritability of functional gradients based on subcortico-cortical functional connectivity remains elusive. Here, leveraging twin functional MRI (fMRI) data from both the Human Connectome Project (n = 1023) and the Adolescent Brain Cognitive Development study (n = 936) datasets, we construct large-scale subcortical functional gradients and delineate an increased principal functional gradient pattern from unimodal sensory/motor networks to transmodal association networks. We observed that this principal functional gradient is heritable, and the strength of heritability exhibits a heterogeneous pattern along a hierarchical unimodal-transmodal axis in subcortex for both young adults and children. Furthermore, employing a machine learning framework, we show that this heterogeneous pattern of the principal functional gradient in subcortex can accurately discern the relationship between monozygotic twin pairs and dizygotic twin pairs with an accuracy of 76.2% (*P* < 0.001). The heritability of functional gradients is associated with the anatomical myelin proxied by MRI-derived T1-weighted/T2-weighted (T1w/T2w) ratio mapping in subcortex. This study provides new insights into the biological basis of subcortical functional hierarchy by revealing the structural and genetic properties of the subcortical functional gradients.

## Abstract (web summary)

An fMRI study on the heritability of human subcortical function, revealing the subcortical functional gradients are heritable, and the strength of heritability exhibits a heterogeneous pattern along a unimodal-transmodal axis.

## Introduction

The human brain, a highly intricate network of anatomically connected and functionally interactive components, constitutes a nested hierarchy supporting perception, cognition, and action^1,2^. Recent advances in human fMRI connectome have brought an analytic tool, i.e., large-scale cortical functional gradients, offering significant insights into the intrinsic dimensions of cortical organization^3,4^. The principal functional gradient on the human cerebral cortex exhibits the unimodal-to-transmodal spatial pattern, tracking a functional hierarchy from direct sensory perception and motor action to the integration and abstraction of information^5^. This spatial transition pattern has been observed to be consistent across species on the cerebral cortex in both human and macaque^6,7^. Moreover, the principle functional gradient was refined with age^8^, correlated with behaviors^9^, linked to gene expression^10^, and disrupted in neurodevelopmental disorders^11^. Despite this critical importance, research on functional gradients and its heritability in the human brain subcortex remains relatively limited.

As a key physiological center, the subcortex supports communication functions and the integration of information under large-scale brain networks^12^. A large amount of studies have demonstrated that the subcortex plays a pivotal role in human behaviors, such as language and memory^13–15^. For example, the thalamocortical pathway supports potential functions of the thalamus in auditory information processing^16^; the striatum is implicated in decision making in both humans and non-human primates^17^; the cerebellum connects with the association cortex and contributes to cognition functions^18,19^. Furthermore, the basal forebrain and midline thalamus have also been substantiated to be associated with widespread variations in fMRI intrinsic cortical activity, serving as key nodes of the ascending neuromodulatory system^20–22^. Clinical studies have concluded that the dysfunction and structural lesions in subcortical regions are considered potential contributors to multiple brain disorders and could influence human behaviors^23–26^. Therefore, performing functional-connectivity gradient analyses of the human subcortex may be beneficial to understand the functional organization of subcortical nuclei and combine with the cerebral cortex as a whole to increase the understanding of complex and adaptive human behaviors.

Besides establishing the spatial heterogeneous features of functional gradients within the human subcortex, it is of paramount importance to delineate the fundamental properties of functional gradients encompassing heritability and anatomical basis. Previous studies have demonstrated that the combination of heritability analyses and neuroimaging methods could serve as a potent approach to understanding how genetic variation may contribute to individual differences in brain structure^27,28^. For example, it has been reported that genetic factors constrain the brain structural phenotypes, including gray matter volume^29–31^, cortical thickness^32,33^ and surface area^29,34^. By utilizing brain functional connectivity, recent advances have studied the genetic influences on the functional organizations of the human cortex^35–39^. More specifically, functional-connectivity-based individualized cortical network topography has demonstrated spatially heterogeneous heritability patterns, revealing a relaxed genetic control of association cortices relative to primary sensory/motor regions based on classical twin design^40^. A recent study by Burger et al.^41^ shed light on the genetic characterization of functional gradients in the cerebral cortex, suggesting that certain aspects of the processing flow from primary regions to network hubs within the default mode network may be under genetic control. Even though multiple studies extensively investigated the heritability of functional organization across the cerebral cortex, only a limited number of them have explored genetic effects on resting-state functional connectivity (RSFC) across cortical and subcortical regions and within the subcortex/limbic system^42,43^. Certain investigations focused on the subcortex have concentrated on the genetic characterization of spatially varying patterns of functional gradients within the hippocampus, revealing substantial genetic mechanisms that regulate functional gradients in specific subregions of the hippocampus^44^. However, the heritability characteristics of the large-scale functional gradients of the entire human subcortical system remain understudied.

In this work, we employed the Laplacian Eigenmap technique, a dimensionality reduction approach, to construct large-scale voxel-wise functional gradients of the subcortico-cortical connectivity for both the Human Connectome Project (HCP) and Adolescent Brain Cognitive Development (ABCD) study individuals. We found functional gradients along the unimodal-to-transmodal axis within the human subcortex for both young adults and children. Specifically, the functional gradients progress along an anterior-to-posterior axis for the hippocampus, along a posterior-to-anterior axis for the thalamus, along an inferior-to-superior axis for the striatum, and along an anterior-to-posterior axis for the cerebellum. Then, we systemically assessed the extent of genetic and environmental contributions to the phenotype of subcortical functional gradients using the ACE model. Our findings revealed that subcortical functional gradients were significantly regulated by genes for both young adults and children. Notably, the heritability of functional gradients was higher in transmodal networks related to unimodal networks across subcortex. Additionally, the heterogeneous pattern of principal functional gradients can discern the relationship between monozygotic (MZ) twin pairs and dizygotic (DZ) twin pairs with an accuracy of 76.2% (*P* < 0.001) utilizing a support vector machine classifier. Lastly, we explored the genetic correlation between the heritability of functional gradients and the architecture of the underlying anatomical myelin represented by T1w/T2w ratio maps in the subcortex. Our results underscored a significant association between the genetic characteristics of functional gradients and T1w/T2w ratio maps.

## Results

We studied the topographical organization and genetic influences on functional gradients within the human subcortex. Our study included 1023 young adults, aged 22–37 years old, from the HCP dataset, and 936 children, aged 9–11 years old, from the ABCD dataset. These data include 125 MZ twin pairs and 74 DZ twin pairs in the HCP dataset, as well as 108 MZ twin pairs and 124 DZ twin pairs in the ABCD dataset. We utilized the Laplacian Eigenmap^45^ techniques for the dimensionality reduction of RSFC data to construct functional gradients within the subcortex in both the HCP and ABCD datasets. For each participant, the RSFC matrix consisted of 1483 regions of interest (ROI) vertices spanning the cortical sheet^46^ and 31,870 voxels within the subcortex, all derived from resting-state fMRI data. We applied the ACE model^47^ on functional gradient 1 (FG1) and functional gradient 2 (FG2) and T1w/T2w ratio to assess the heritability. Furthermore, we evaluated whether the similarity of the FG1 could be used to classify the relationships between MZ twin pairs and DZ twin pairs in the HCP dataset. Additionally, we leveraged subcortical myelin proxied by the T1w/T2w ratio to establish the relationship of heritability between human subcortical function and structure.

### Large-scale functional gradients of subcortex in young adults and children

**Fig. 1:**
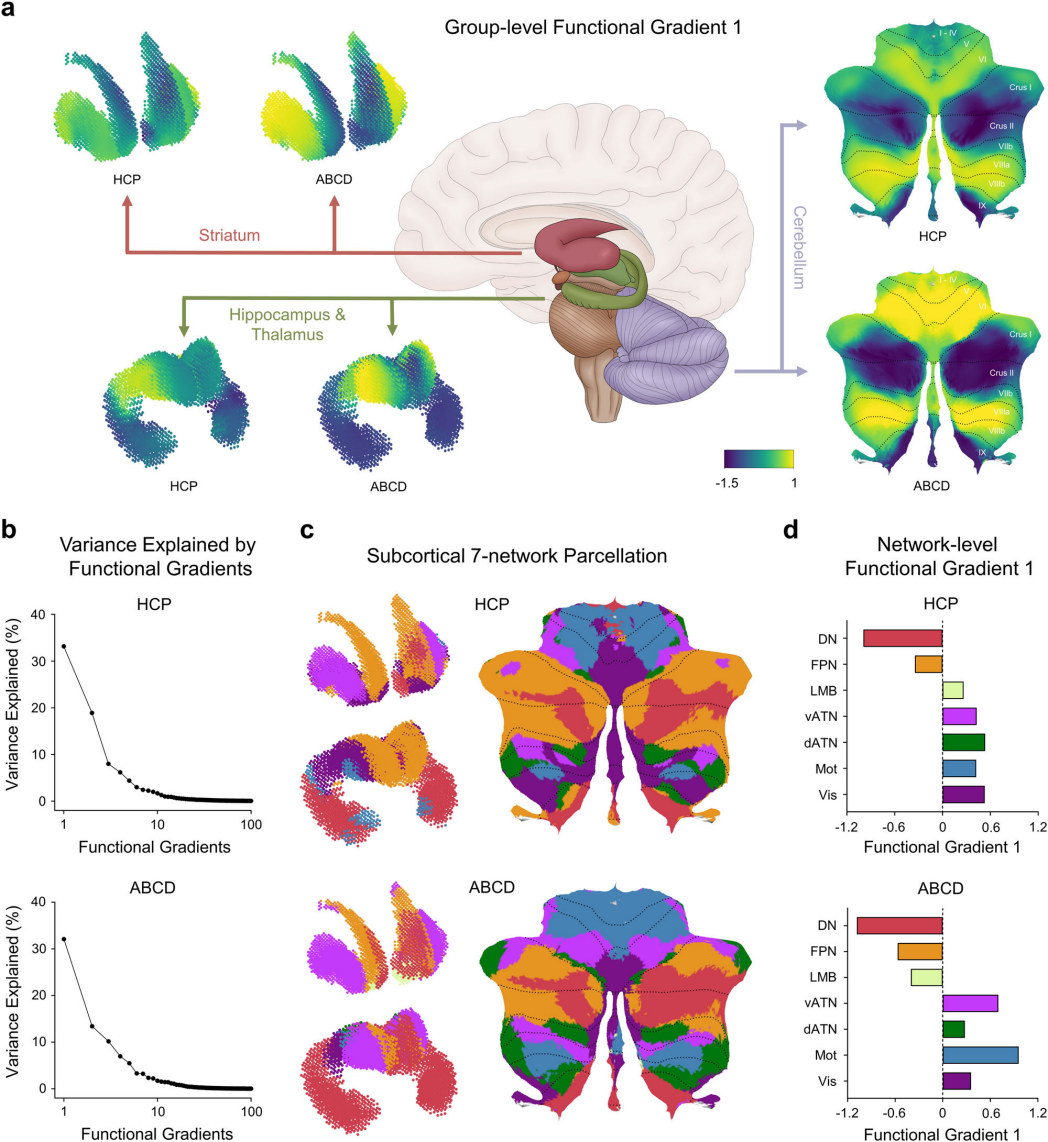
Group-level functional gradient 1 of subcortex for the HCP and ABCD datasets. **a** Group-level functional gradient 1 (FG1) in the striatum, hippocampus, thalamus, and cerebellum for the HCP and ABCD datasets. **b** Variance explained for the HCP (upper) and ABCD (lower) functional gradients of the subcortex, respectively. The HCP and ABCD group-level FG1 and FG2 account for more than 40% of the variance. **c** Subcortical 7-network parcellation of the HCP and ABCD datasets, respectively. For visualization, only four main subareas are presented, including the striatum, hippocampus, thalamus, and cerebellum. The delineations of the 7-network parcellation of subcortex are based on an optimized winner-take-all strategy (please see “Methods” section for more details). **d** Network-level distribution of the FG1 values is highly consistent between the HCP (upper) and ABCD (lower) datasets. FG1 values of both the HCP and ABCD datasets exhibit a transition gradient from unimodal-to transmodal networks. DN default mode network, FPN frontoparietal network, LMB limbic network, vATN ventral attention network, dATN dorsal attention network, Mot somatomotor network, Vis visual network.

Group-level FG1 for the HCP and ABCD datasets exhibit significant similarity within the subcortex (Spearman’s *r* = 0.819, *P*_moran_ < 0.001), including striatum, thalamus, hippocampus, and cerebellum (Fig. 1a). This substantial similarity in spatial distribution of FG1 between young adults and children implies that the spatial organization of the subcortico-cortical interactions has achieved relatively stable state during childhood. The first two gradients, which explain the majority of variance in functional connectivity, slightly differed between the HCP (Fig. 1b, upper) and ABCD (Fig. 1b, lower) datasets. Specifically, FG1 accounted for 33.2% of the variance in the HCP group-level RSFC and 32.1% of the variance in the ABCD group-level RSFC; FG2 accounted for 18.9% of the variance in the HCP group-level RSFC and 13.4% of the variance in the ABCD group-level RSFC. Consequently, the subsequent analyses of topography and heritability in functional gradients primarily focused on the first and second functional gradients.

The group-level FG1 exhibits spatial heterogeneity within the subcortex. In the cerebellum, the description of spatial anatomical location for FG1 is based on the probabilistic lobular atlas^48,49^ including V, VI, crus I, crus II, VIIIa, and VIIIb. In the cerebellum, the FG1 is anchored at one end by V, VI, VIIIa, VIIIb, and at the other end by crus I, and crus II, indicating an anterior-to-posterior transition (Fig. 1a, right). In the striatum, FG1 exhibits an inferior-to-superior transition, extending from the caudate nucleus to the putamen (Fig. 1a, left top). In the hippocampus and thalamus, a continuous transition begins in the anterior thalamus, passes through the posterior thalamus and posterior hippocampus, and ends in the anterior hippocampus, with the peak FG1 values situated at the middle and posterior thalamus (Fig. 1a, left bottom). The precise location of the shift differs slightly between young adults and children in the hippocampus and thalamus. Specifically, the center of peak values shifts from the middle thalamus to the posterior thalamus and posterior hippocampus in young adults compared to children. Additionally, the group-level FG2 between young adults and children also contains obvious differences in spatial pattern (Supplementary Fig. 1). We noted that in children, the peak values of FG2 are located in the nucleus accumbens (at the front of the striatum), while in young adults, higher gradient values are found in the middle putamen and the tail of the caudate. Furthermore, the low values of FG2 in the hippocampus and thalamus locate in the anterior hippocampus in young adults, but in the posterior thalamus in children. In the cerebellum, there exists an inconsistent transition of the FG2 values between young adults and children, particularly in crus I, crus II, VIIb, VIIIa, VIIIb, and IX. For more detailed results of functional hierarchy analyses, please refer to Supplementary Fig. 1.

Last, we investigated whether the transition of functional gradients within the subcortex corresponded to the hierarchy of functional networks in the cerebral cortex. Subcortical functional network parcellations, based on 7-network parcellation on the cortical sheet^50^, were defined using a similar procedure as in prior studies^51–53^ for both the HCP (Fig. 1c, upper) and ABCD (Fig. 1c, lower) datasets, along with their confidence maps (Supplementary Fig. 2a, b) providing more convincing parcellation results^52^. These networks are broadly categorized into two main groups: unimodal networks, including somatomotor network (Mot) and visual network (Vis), and transmodal association networks, including default mode network (DN), frontoparietal network (FPN), ventral attention network (vATN), dorsal attention network (dATN) and limbic network (LMB)^54^. Based on these functional network parcellations, we evaluated the association between functional gradients and the hierarchy of functional networks. We found a gradient transition for FG1 from the unimodal networks to transmodal networks in the subcortex for both the HCP (Fig. 1d, upper) and ABCD (Fig. 1d, lower) datasets. Additionally, we examined the functional hierarchy of functional gradients within each subcortical nuclei, including the striatum, hippocampus, thalamus, and cerebellum. Specifically, the FG1 in transmodal networks is also different from those in unimodal networks in young adults (Supplementary Fig. 3a) and children (Supplementary Fig. 3b). These findings suggest that functional gradients in subcortex exhibit a spatially heterogeneous pattern aligning with the functional hierarchy in the cerebral cortex^3^, and show high consistency in both young adults and children.

### Individual functional gradients of the subcortex are more similar in monozygotic twins than in unrelated individuals

**Fig. 2:**
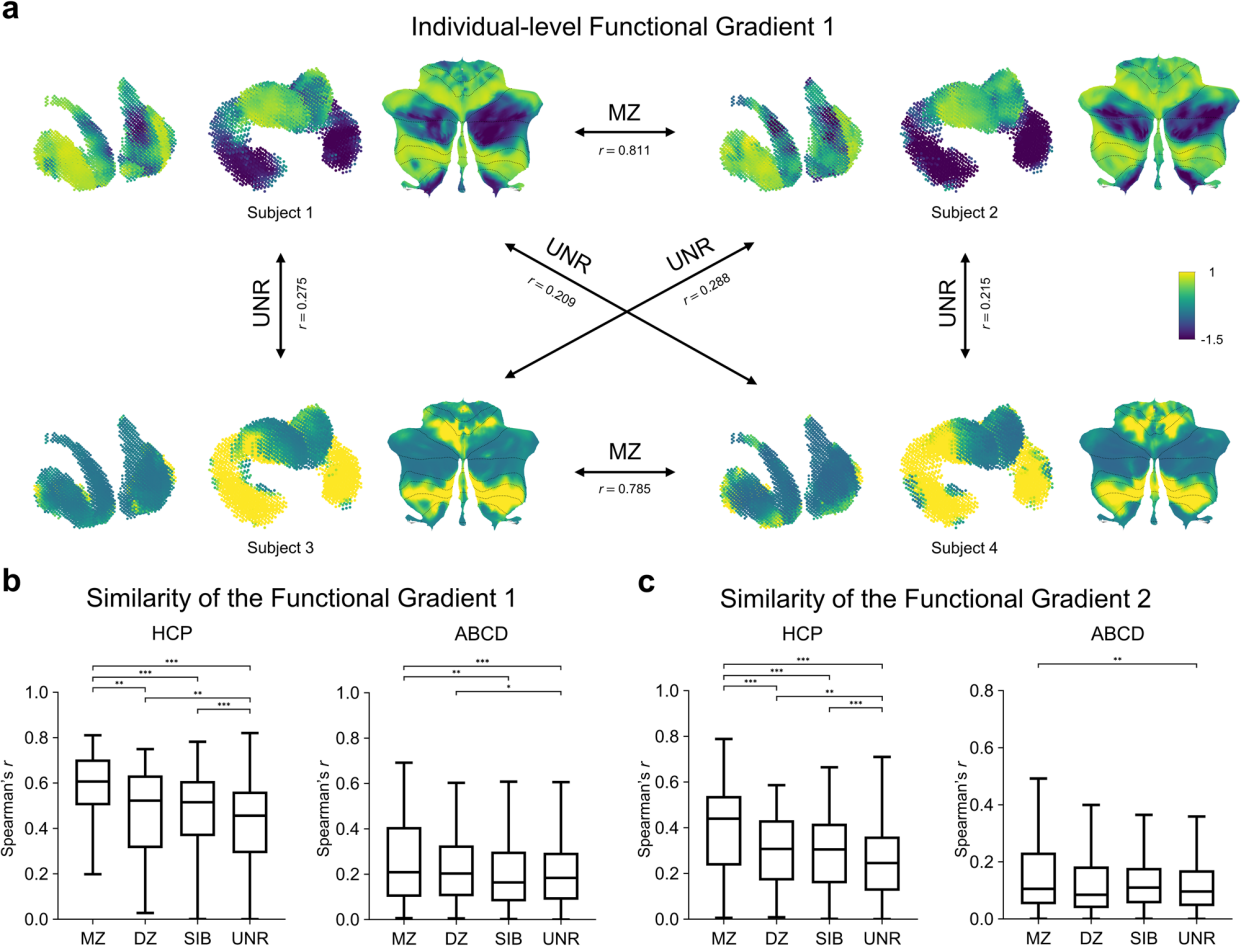
Individual-level FG1 maps are more similar within twins relative to siblings and unrelated individuals in both the HCP and ABCD datasets. **a** The spatial maps of FG1 for four exemplary individuals exhibit greater differences between UNR pairs, while marked similarities between MZ twin pairs in the HCP dataset. The spatial similarity is represented by using Spearman correlation (subject 1, subject 2, subject 3 and subject 4 correspond to the HCP subject IDs of HCP181232, HCP191841, HCP104416, and HCP187345). **b** The spatial similarity of FG1 among MZ twin pairs, DZ twin pairs, SIB pairs, and UNR pairs in both the HCP (left) and ABCD (right) datasets. **c** The spatial similarity of FG2 among MZ twin pairs, DZ twin pairs, SIB pairs, and UNR pairs in both the HCP (left) and ABCD (right) datasets. These results demonstrate that higher similarity of functional gradients among MZ, relative to DZ, SIB, and UNR pairs. MZ monozygotic twins, DZ dizygotic twins, SIB siblings, UNR unrelated individuals; ^*^P < 0.05, ^**^P < 0.01, ^***^P < 0.001, two-tailed two-sample *t*-test.

Having evaluated the subcortical functional gradients at the group level, we next performed the Laplacian Eigenmap technique to individualized functional-connectivity matrix and constructed individualized functional gradients for both the HCP and ABCD datasets. We aligned individualized functional gradients to the group-level functional gradients of the HCP and ABCD datasets, respectively, and next investigated the spatial similarity of functional gradients across different kinship relationships. The FG1 exhibits a higher degree of spatial similarity between MZ twin pairs relative to unrelated individuals in the HCP dataset (Fig. 2a). Then, we further quantified the spatial similarity of FG1 in both the HCP (Fig. 2b, left) and ABCD (Fig. 2b, right) datasets, as well as FG2 in the HCP (Fig. 2c, left) and ABCD (Fig. 2c, right) datasets within the subcortex among different kinship relationship pairs. Quantitative analyses revealed that FG1 and FG2 exhibited greater spatial similarity among relatives compared to unrelated individuals in both the HCP and ABCD datasets. Specifically, the similarity of the FG1 and FG2 are significantly higher for MZ twin pairs relative to DZ twin pairs, siblings (SIB) pairs, and unrelated individual (UNR) pairs in the HCP datasets (two-tailed two-sample *t*-test, all *P*-values < 0.01). Similarly, in the ABCD dataset, both FG1 and FG2 present higher spatial similarity in MZ twin pairs relative to UNR pairs (two-tailed two-sample *t*-test, all *P*-values < 0.01). These results suggest that the spatial organization of functional gradients in the human subcortex may be influenced by genetic factors.

### Heritability of functional gradients presents a spatial heterogeneous distribution

**Fig. 3:**
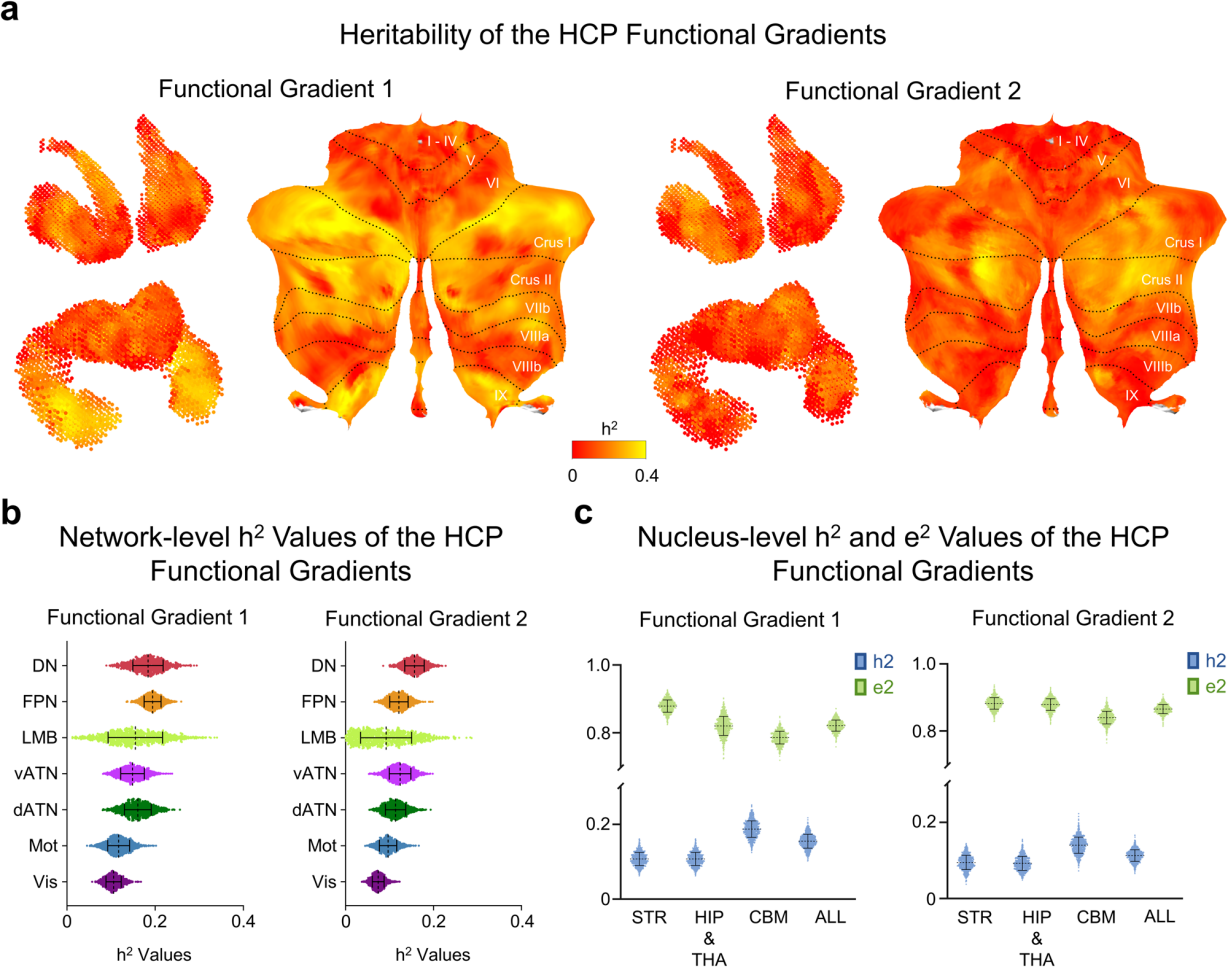
Heritability of the HCP FG1 and FG2 in subcortex. **a** Heritability maps of FG1 (left) and FG2 (right) in the striatum, thalamus, hippocampus, and cerebellum in the HCP dataset. **b** Network-level heritability of FG1 (left) and FG2 (right) in the HCP dataset represented by the *h*^2^ index exhibit a decreasing pattern from transmodal to unimodal networks. Across seven canonical networks, the frontoparietal and default mode networks show higher heritability relative to other networks in the FG1 and FG2, respectively. Distribution of data points was calculated through bootstrap with 1000 times (please see “Methods” section for more details). **c** The additive genetic and unique environmental contribution to the FG1 (left) and FG2 (right) in different subcortex of the HCP dataset. Compared with the striatum, hippocampus, and thalamus, genetic contribution in the cerebellum is higher for both the FG1 and FG2. The contribution of a unique environment is highest in the striatum for both the FG1 and FG2. Distribution of data points was calculated through bootstrap with 1000 times. ALL whole subcortex, STR striatum, HIP & THA hippocampus and thalamus, CBM cerebellum.

To objectively examine the degree of genetic influences on human subcortical functional gradients, we employed Accelerated Permutation Inference for the ACE model^47^ to estimate genetic effects (A), common environmental effects (C), and unique environmental effects (E) in voxel-wise subcortical functional gradients. Heritability estimations on functional gradients in the HCP dataset revealed that the FG1 (*h*^2^: Mean = 0.155, SD = 0.019, Fig. 3a, left) and FG2 (*h*^2^: Mean = 0.113, SD = 0.015, Fig. 3a, right) are controlled by genetic effects. We conducted likelihood ratio tests (LRT) to assess the significance of heritability for FG1 and FG2 in the HCP dataset. The results demonstrated that the transmodal networks in FG1 and FG2 were significantly controlled by inherited genetics in the HCP dataset (Supplementary Fig. 4). In the ABCD dataset, heritability estimates for both FG1 (*h*^2^: Mean = 0.049, SD = 0.008, Supplementary Fig. 5a) and FG2 (*h*^2^: Mean = 0.034, SD = 0.004, Supplementary Fig. 5b) were relatively low and did not meet the criteria of statistical significance with false discovery rate (FDR) correction. Furthermore, there exists a significant spatial correlation between the spatial distribution of the heritability of FG1 in the HCP and ABCD datasets (Spearman’s *r* = 0.213, *P*_moran_ < 0.001). These results indicate that the heritability patterns of the functional gradient in the subcortex are similar between children and young adults. The estimations of the common environmental effects and unique environmental effects were also conducted (Supplementary Fig. 6). The spatial maps of *e*^2^ exhibit a similar but reverse pattern to the heritability estimations, while those of *c*^2^ are relatively sparse and random. We did not perform comparative analyses on the absolute value of the functional gradients and their heritability between these two datasets. Instead, we focused on the spatial transitional traits. Subcortex with high genetic contributions include crus I, crus II, VIIb, IX in the cerebellum, anterior hippocampus, middle putamen, and middle caudate in both FG1 and FG2 heritability in the HCP dataset. Moreover, we evaluated the genetic correlations (rG) between the FG1 and FG2 using the umx toolbox^55^, which is a user-friendly interface based on the OpenMx toolbox. Voxels that have passed the LRT in univariate analyses of both the FG1 and FG2 were used in calculating genetic correlations. Therefore, we only evaluated the genetic correlations in the HCP datasets since the heritability estimates of both FG1 and FG2 in the ABCD dataset did not meet the criteria of statistical significance. The results show that there exist strong genetic correlations between FG1 and FG2 in the HCP (Mean_|rG|_ = 0.512, SD_|rG|_ = 0.341, Supplementary Fig. 7a, left). The mean absolute values of genetic correlations are relatively close between the striatum and cerebellum in the genetic correlation between HCP FG1 and HCP FG2 while these values are lower in the hippocampus and thalamus (Supplementary Fig. 7a, right).

After evaluating the global heritability of the whole subcortex, we proceeded to quantify local heritability within each functional network parcellations. Specifically, the *t*-tests of functional gradient heritability across networks used the bootstrap strategy to establish a statistical index and averaged bootstrap measurements across unimodal-transmodal networks to conduct a two-sample *t*-test (see “Methods” section for details). Our findings show that the heritability estimations exhibit non-uniform distribution across subcortical functional networks, with the greatest heritability observed within transmodal networks compared to unimodal networks for both FG1 (F (6, 6993) = 918.7, *P* < 0.0001; two-tailed two-sample *t*-test between unimodal and transmodal, *P* < 0.0001, Fig. 3b, left) and FG2 (F (6, 6993) = 831.6, *P* < 0.0001; two-tailed two-sample *t*-test between unimodal and transmodal, *P* < 0.0001, Fig. 3b, right) in the HCP dataset. This pattern also holds for FG1 (F (6, 6993) = 1099, *P* < 0.0001; two-tailed two-sample *t*-test between unimodal and transmodal, *P* < 0.0001, Supplementary Fig. 5c) in the ABCD dataset. In contrast, the heritability of FG2 in the ABCD dataset does not present a significant difference between unimodal and transmodal networks (F (6, 6993) = 921.6, *P* < 0.0001; two-tailed two-sample *t*-test between unimodal and transmodal, *P* = 0.130, Supplementary Fig. 5d). These findings indicate that the heritability of subcortical FG1 and FG2 follows a hierarchy along the unimodal-transmodal axis. Additionally, the heritability also varies across different subcortical subareas, with the mean heritability of FG1 (Fig. 3c, left) and FG2 (Fig. 3c, right) exhibiting both the highest in cerebellum in young adults. Nevertheless, the proportion of genetic contribution did not show much difference across different subcortical subareas in children (Supplementary Fig. 5e, f). To be noticed, environmental effects still contribute crucially to phenotypic variances in functional gradients in both young adults and children.

It is still important to mention that there exist multiple differences between the HCP and ABCD datasets, including but not limited to data quality and multi-site/multi-scanner effects. To assess whether these differences would impact our conclusions regarding the heritability estimations, we estimated SNR represented by Signal-to-Fluctuation-Noise Ratio (SFNR) (Supplementary Fig. 8) and analyzed associations between the heritability estimations and the SNR. Although there exist significant correlations between SNR and heritability in the HCP dataset (SFNR – FG1 *h*^2^: Spearman’s *r* = 0.483, *P*_moran_ < 0.001; SFNR – FG2 *h*^2^: Spearman’s *r* = 0.498, *P*_moran_ < 0.01), no significant associations have been discovered in the ABCD dataset (SFNR - FG1 *h*^2^: Spearman’s *r* = -0.007, *P*_moran_ = 0.4; SFNR – FG2 *h*^2^: Spearman’s *r* = 0.044, *P*_moran_ = 0.117). The results suggest that our heritability estimations do not simply reflect data quality. However, better data quality would help the heritability estimation procedure capture more detailed differences between the relatives and the unrelated individuals, leading to higher estimation values. Moreover, considering the potential impacts of the multi-site/multi-scanner effects, we have also validated the heritability estimations of FG1 and FG2 on the ABCD dataset with balanced sites. This balanced subset consisted of 444 participants from 3 sites equipped with Siemens Prisma MR scanner, which were further selected from the initial discovery set. Subsequently, we computed the heritability estimates for both the FG1 (*h*^2^: Mean = 0.047, SD = 0.007, Supplementary Fig. 9a) and FG2 (*h*^2^: Mean = 0.040, SD = 0.005, Supplementary Fig. 9c) in the balanced subset. The heritability estimations significantly correlate between the discovery set and balanced subset for both the FG1 (Spearman’s *r* = 0.650, *P*_moran_ < 0.001, Supplementary Fig. 9b) and FG2 (Spearman’s *r* = 0.638, *P*_moran_ < 0.001, Supplementary Fig. 9d). Although the heritability of the balanced subset was not entirely identical to the discovery set, this result still indicated that the multi-scanner effect would not substantially alter the heritability estimations in the ABCD discovery set. Moreover, the heritability estimations are relatively stable after regressing out nuisance variables, including ${age},{sex},{age}\times {sex},{{age}}^{2}$ and ${{age}}^{2}\times {sex}$ (Supplementary Fig. 10). The results are highly similar between the heritability estimations with and without nuisance regressions (HCP FG1: Spearman’s *r* = 0.957; HCP FG2: Spearman’s *r* = 0.965; ABCD FG1: Spearman’s *r* = 0.981; ABCD FG2: Spearman’s *r* = 0.975).

### Functional gradient-based machine learning classifier could identify the difference between MZ twin pairs and DZ twin pairs

**Fig. 4:**
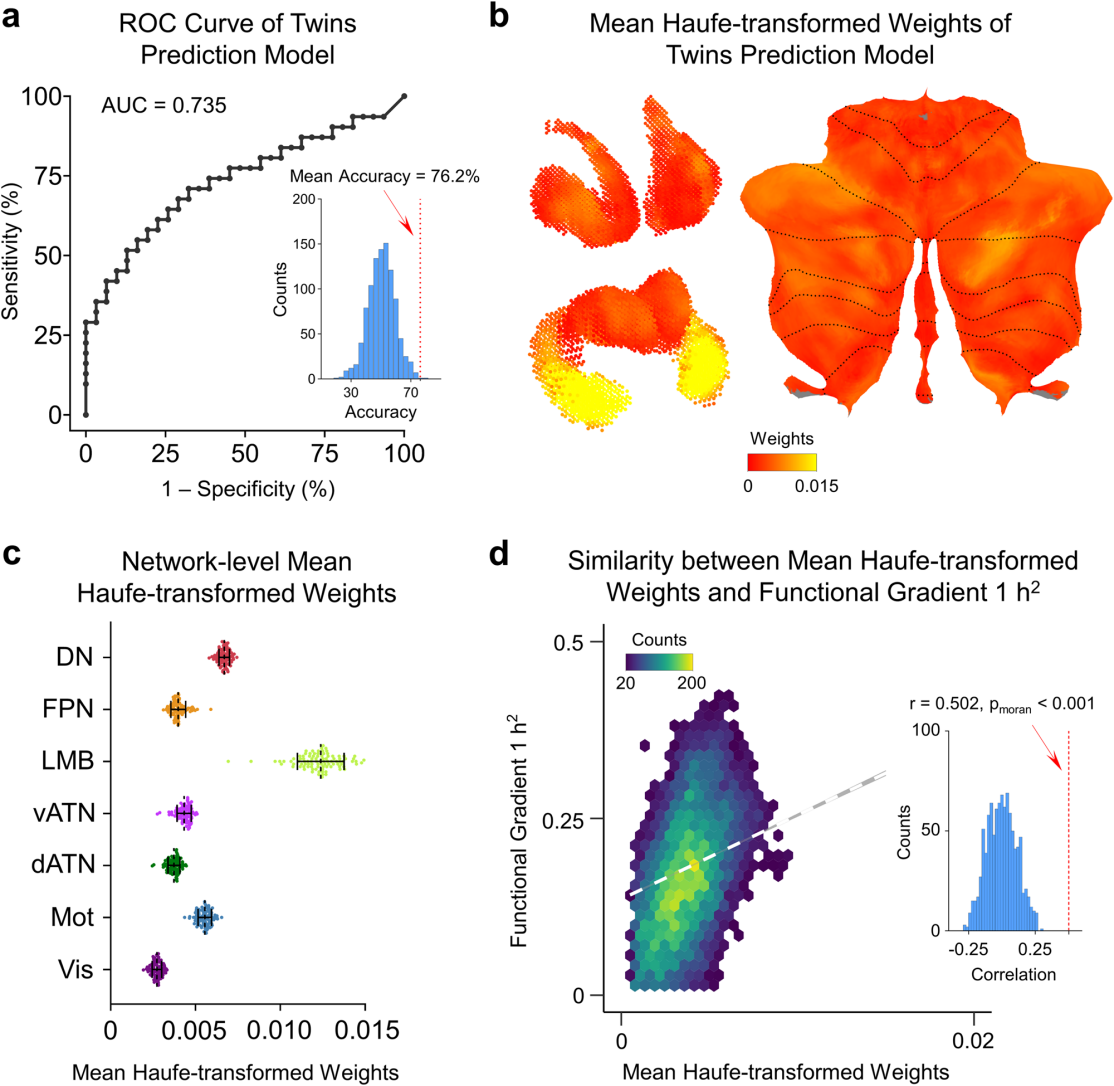
The FG1 could help to classify MZ twin pairs and DZ twin pairs in the HCP dataset. **a** Receiver operating characteristic (ROC) curve of the twin pair classification model. The area under the ROC curve is 0.735 and the model classified the relationship of individual pairs as MZ twin pairs and DZ twin pairs as mean classification accuracy reaches 76.2% (*P* < 0.001); the inset histogram represents the mean actual classification accuracy (red dashed line) and the null distribution generated from the permutation test with 1000 times. **b** The mean spatial map of Haufe-transformed weights. These weight maps exhibit a heterogeneous pattern across the human subcortex, with the higher values indicating more contributions in distinguishing between MZ twin pairs and DZ twin pairs. **c** The values of the Haufe-transformed weight maps are summed across 7-network parcellations in the subcortex. The weight values in transmodal networks are significantly higher than that in unimodal networks (F (6, 693) = 2715, *P* < 0.0001; two-tailed two-sample *t*-test between unimodal and transmodal, *P* < 0.0001), which demonstrates FG1 in transmodal networks contribute the most in the classification model. Distribution of data points was obtained through 100 repeated classifications (please see “Methods” section for more details). **d** The spatial maps of the summed Haufe-transformed weights were associated with the strength of *h*^2^ maps of FG1 in the HCP dataset (Spearman’s *r* = 0.502, *P*_moran_ < 0.001); the inset histogram represents the actual spatial correlation (red dashed line) compared with the null distribution generated from the Moran Spectrum Randomization strategy.

Having quantified the genetic control of functional gradients in the human subcortex, we further examined whether a machine learning classifier could discriminate between MZ twin pairs and DZ twin pairs. We leveraged individualized FG1 from the HCP dataset to train a Gaussian kernel support vector machine (SVM) classifier. We leveraged randomly selected 74 of the 125 MZ twin pairs in the HCP dataset and the complete set of the 74 DZ pairs for training and testing. The participants’ age, sex, and in-scanner head motion were accounted for as covariates regressed by SurfStat Toolbox (https://math.mcgill.ca/keith/surfstat/) with a general linear model (GLM). After 100 repeated five-fold cross-validation (5F-CV) procedures, the classifier achieved a mean accuracy of 76.2% (permutation test *P* < 0.001, Fig. 4a). The area under the mean receiver operating characteristic (ROC) curve is 0.735 (permutation test *P* < 0.001). We have also trained other six SVM classifiers involving the siblings and unrelated individuals, namely (1) SIB – MZ classifier (ACC = 64.0%, AUC = 0.682), (2) SIB – DZ classifier (ACC = 52.6%, AUC = 0.506), (3) SIB – UNR classifier (ACC = 53.3%, AUC = 0.588), (4) UNR – MZ classifier (ACC = 66.4%, AUC = 0.674), (5) UNR – DZ classifier (ACC = 56.2%, AUC = 0.572) and (6) UNR – Twins (MZ and DZ) classifier (ACC = 61.9%, AUC = 0.638). The results show that the accuracies of these classifiers are relatively lower than the MZ – DZ classifier (Supplementary Fig. 11).

To comprehend the gradient’s contribution to SVM classification, we selected the MZ – DZ classification model since the heritability estimations were obtained from the difference of magnitude between MZ twin pairs and DZ twin pairs^47,56^, and inverted it into a forward model^57,58^. Haufe’s inversion approach yields a predictive feature map, either positive or negative, referred to as the Haufe-transformed weight map, for the SVM weight map. Higher values in the Haufe-transformed weight map signify a greater contribution to classifying kinship relationships. We observed that the mean Haufe-transformed weight map exhibited a heterogeneous pattern across subcortex (Fig. 4b). To further evaluate which functional network contributes the most to the classification, the absolute values of the Haufe-transformed weight map were aggregated across 7-networks parcellations (Fig. 4c). These results indicated that transmodal networks, for example, FPN and DN, contribute more to the classification model. Moreover, for the mean Haufe-transformed weight obtained from the 100 times 5F-CV SVM model, we conducted statistical analyses comparing the average weights in transmodal versus unimodal networks. The result revealed a significant difference in Haufe-transformed weights between the transmodal and unimodal networks (F (6, 693) = 2715, *P* < 0.0001; two-tailed two-sample *t*-test between unimodal and transmodal, *P* < 0.0001), which aligned with our findings on heritability estimations of functional gradients in human subcortex. Then, we assessed the similarity between the results of the Haufe-transformed weight map and the *h*^2^ map for FG1 in the HCP dataset. The mean Haufe-transformed weight map was significantly correlated with the *h*^2^ map for FG1 (Spearman’s *r* = 0.502, *P*_moran_ < 0.001).

### Genetic correlation between individualized subcortical functional gradients and T1w/T2w ratio

**Fig. 5:**
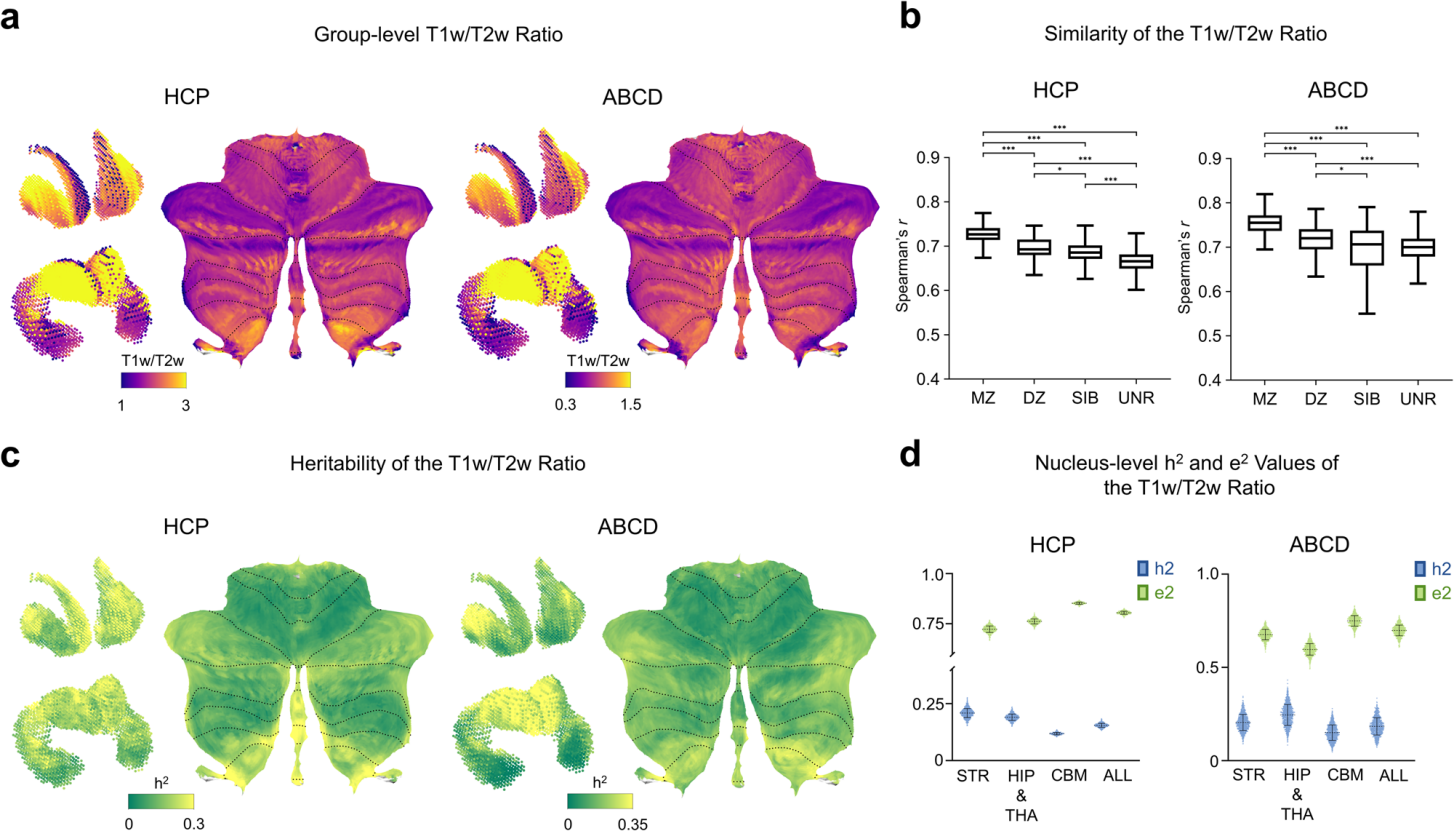
The heritability pattern of an anatomical representation proxied by T1w/T2w ratio in both the HCP and ABCD datasets. **a** The group-level T1w/T2w ratio maps between HCP (left) and ABCD (right) datasets exhibit significant spatial correlation (Spearman’s *r* = 0.949, *P*_moran_ < 0.001). **b** The spatial similarity of the T1w/T2w ratio within MZ twin pairs is significantly higher than DZ twin pairs (two-tailed two-sample *t*-test, *P* < 0.001), SIB (two-tailed two-sample *t*-test, *P* < 0.001) and UNR pairs (two-tailed two-sample *t*-test, *P* < 0.001) for both the HCP (left) and ABCD (right) datasets. ^*^P < 0.05, ^**^P < 0.01, ^***^P < 0.001, two-tailed two-sample *t*-test. **c** Heritability maps of T1w/T2w ratio in the striatum, thalamus, hippocampus, and cerebellum in the HCP (left) and ABCD (right) datasets. **d** The additive genetic and unique environmental contribution to the T1w/T2w ratio in the subcortex of the HCP (left) and ABCD (right) datasets, respectively. Genetic contribution in the striatum is higher for the T1w/T2w ratio compared with the hippocampus, thalamus, and cerebellum in the HCP dataset. In the ABCD dataset, the highest genetic contribution focuses on the hippocampus and thalamus. The contribution of a unique environment is highest in the cerebellum for the T1w/T2w ratio in both the HCP and ABCD datasets. Distribution of data points was calculated through bootstrap with 1000 times (please see “Methods” section for more details). ALL whole subcortex, STR striatum, HIP & THA hippocampus and thalamus, CBM cerebellum.

As a proxy for the underlying fundamental organization of the human brain structure, the gradients of myelin maps exhibit remarkable alignment with the gradients of published probabilistic cytoarchitectonically defined cortical areas^59^. In this study, we selected myelin, approximated by the T1w/T2w ratio, as a representation of the fundamental organization of the subcortex. We aimed to understand whether the heritable architecture of functional gradients in the subcortex is related to the gene-controlled myelin phenotypes. Subsequently, we constructed T1w/T2w ratio maps for young adults and children, which serve as indicators of myelination development, and employed the ACE model to estimate its heritability. First, we delineated group-averaged T1w/T2w ratio maps in human subcortex. These maps exhibited significant correlation with FG1 in both the HCP (Spearman’s *r* = 0.238, *P*_moran_ < 0.05, Supplementary Fig. 12, left) and ABCD (Spearman’s *r* = 0.274, *P*_moran_ < 0.05, Supplementary Fig. 12, right) datasets. In contrast to FG1 and FG2, the spatial patterns of two group-level T1w/T2w ratio maps (Fig. 5a) are highly similar (Spearman’s *r* = 0.949, *P*_moran_ < 0.001). In consistent with the findings in functional gradients, stronger spatial correlations could also be discovered between individualized T1w/T2w ratio maps of MZ twin pairs, DZ twin pairs and SIB pairs than those of UNR pairs in both the HCP and ABCD datasets (two-tailed two-sample *t*-test, all *P*-values < 0.001, Fig. 5b). These results offer substantial evidence supporting the heritability of T1w/T2w ratio maps in human subcortex. Then, analyses of the ACE model denoted that interindividual differences in T1w/T2w ratio maps were indeed influenced by inherited genetics in both the HCP (*h*^2^: Mean = 0.155, SD = 0.009, Fig. 5c, left) and ABCD (*h*^2^: Mean = 0.184, SD = 0.046, Fig. 5c, right) datasets. Following the significance testing using the LRT, it was evident that T1w/T2w ratio maps in transmodal networks were significantly controlled by genes (Supplementary Fig. 13).

We then probed whether there are genetic correlations (rG) between functional gradients and T1w/T2w ratio. Voxels that have passed the LRT in univariate genetic analysis of both FG1 and T1w/T2w ratio were included in calculating genetic correlations of T1w/T2w ratio and FG1. A similar strategy was used in calculating genetic correlations of the T1w/T2w ratio and FG2. Therefore, we only evaluated the genetic correlations in the HCP datasets since the heritability estimates of both FG1 and FG2 in the ABCD dataset did not meet the criteria of statistical significance. The results exhibited strong genetic correlations in young adults (T1w/T2w ratio versus FG1: Mean_|rG|_ = 0.778, SD_|rG|_ = 0.387, Supplementary Fig. 7b, left; T1w/T2w ratio versus FG2: Mean_|rG|_ = 0.342, SD_|rG|_ = 0.332, Supplementary Fig. 7c, left). The mean absolute values of genetic correlations are relatively close among the striatum, hippocampus, thalamus, and cerebellum in both genetic correlation analyses (Supplementary Fig. 7b, c, right). Additionally, similar to the findings in the heritability of functional gradients, the heritability of the T1w/T2w ratio also varies across different subcortex. Specifically, the genetic contribution to T1w/T2w in the cerebellum is lower compared to that in the striatum, the hippocampus, and thalamus in both datasets (Fig. 5d). However, this heritable architecture of anatomical structure was opposite to heritability estimation results in functional gradients, where the heritability of FG1 and FG2 are both highest in the cerebellum. These results suggested that both the anatomical T1w/T2w ratio maps and functional gradients followed similar spatial axis as well as their heritability but in an opposite pattern^6^.

## Discussion

The subcortex plays a pivotal role in cognitive, affective, and social functions through supporting communication functions and the integration of information under large-scale brain networks in humans^60–63^. Previous studies focused on interactions between part of subcortical and cortical regions, employing state-of-the-art neuroimaging techniques, which have made significant strides in comprehending the fundamental organization of the subcortex^64,65^. In the current study, we systematically investigated the topography of the human subcortex in two extensive twin-sample datasets, including the HCP and ABCD. Our result revealed that the functional gradients exhibited a clear spatially heterogeneous pattern within the subcortex, which is similar between young adults and children. Specifically, the FG1 was anchored at one end by transmodal association networks, corresponding in humans to the DN, and at the other end by unimodal networks. It should be noted here, that unimodal and transmodal networks were inherited from the cortical terminology through mapping the cortical 7-network parcellation into the subcortex. Then, we demonstrated that functional gradients in the subcortex are heritable, which pronounced a large genetic effect on higher-order transmodal relative to unimodal networks in both FG1 and FG2. Additionally, we utilized a functional gradient-based machine learning classifier, which could identify kinship relationships between MZ twin pairs and DZ twin pairs with an accuracy of 76.2% (*P* < 0.001). Lastly, we uncovered a genetic correlation between subcortical FG1 and T1w/T2w ratio. These results shed new light on the topography and heritability of functional gradients in the human subcortex, suggesting a neuroanatomical basis behind the human brain subcortical function gradients.

Using two large twin samples datasets for young adults and children, we found reproducible results in the topography and heritability of subcortical functional gradients. Notably, the spatial heterogeneity of the functional gradients represented the largest successful replication, even though there exists multiple differences between these two datasets: scanner hardware, parameters of the imaging sequences, and the length of resting-state fMRI data, which fully demonstrates the robustness and stability of the results of this study. Besides, the heritability of FG1 also exhibits a similar hierarchical pattern along a unimodal-transmodal axis between young adults and children. These remarkably consistent findings across datasets may be attributed to several important factors. First, both these two datasets leveraged a previously unprecedented number of fMRI scans of adults (*n* = 1023) and children’s (*n* = 936) brains, which provided sufficient power to uncover reliable functional gradients and its corresponding heritability. Second, the high quality of precision functional brain mapping and state-of-the-art dimensionality reduction approach enabled us to harness the interindividual variability in functional gradients as valuable information, rather than disregarding it as noise. Third, large-scale functional gradients were decomposed from functional connectivity, capturing higher-level and more predominant features of the brain functions, which would mitigate the impact of differences in data quality between the HCP and ABCD datasets.

Previous studies in the adult cerebral cortex have illustrated the presence and topological features of a gradient of functional brain connectivity from the primary sensory cortex to associative cortical areas^3,4^. Simultaneously these functional gradients exhibit precise responsive perceptual performance and abstract cognitive functions^9^. Other studies have revealed that the development of functional gradients is mediated by the developmental changes in network integration and segregation, which are associated with high-order cognitive functions^8,10^. A longitudinal study of functional gradients across the lifespan detected a strong shift of the visual network’s position from an extreme to a more central position across the adult lifespan, while the transmodal networks remained stable but increasingly dispersed with age, showing a negative association with cognitive functions^66^. Other studies about functional gradients in neonates have demonstrated that the primary neonatal connectome gradient spans between sensorimotor and visual anchors^67,68^. However, studies involving the core large-scale functional gradients within the subcortex in both young adults and children were still insufficient. Our study extended previous studies by establishing an architecture of large-scale functional gradients in the subcortex based on subcortico-cortical RSFC in both young adults and children and demonstrated their functional transitions at the network level.

Most previous publications have focused their investigations on understanding genetic factors contributing to cortical phenotypes including anatomy and function. For example, studies on the heritability of brain anatomy have indicated that multiple anatomical features^69,70^ including cortical surface area^71^, thickness^32^, cortical folds^72^, and volume^73^ are controlled by genetic effects. Similarly, concerning cortical functions, genetic contributions have also been observed to influence functional connectivity^74,75^, networks^38,39,76,77^, and topography^40^. Although plenty of genetic studies have concentrated on cortical regions, investigations into how genes regulate subcortex and functions have often been overlooked. Given that the subcortex serves as crucial hubs along the pathway from the spinal cord to the cerebral cortex, understanding the heritability of these structures would provide us with a new perspective on how genes and environment influence individual cognitive, affective, and social functions. In recent years, research endeavors have begun to investigate the heritability of subcortical anatomies including brain volumes^78,79^ and shape^31,80^. Other studies have investigated the heritability of subcortical functional connectivity as a part of ROI-based whole-brain study^36,37,81^, and delineated the gene expression pattern in cortico-striatal regions^42^. Even fewer studies demonstrated that some certain subcortical nuclei, including hippocampus^44^ and ventral striatum^43^, are functionally heritable on a finer scale. However, there is still a lack of studies probing the heritability features of the subcortex in a unified and fine-grained vision. Regarding subcortical regions as a whole, we observed substantial spatial heterogeneity in the influence of genetic factors across the subcortex. Through 7-network parcellations in the subcortex, we quantified the heritability difference across different functional networks in both the HCP and ABCD datasets. Heritability estimations on functional gradients show a clear decrease from transmodal networks to unimodal networks in these two datasets. In the human brain, transmodal networks play an important role in higher-order cognition^82^, while unimodal networks are responsible for sensorimotor functions. Thus, our results of the heritability estimations also suggested a gradual loosening of genetic control from cognition to sensation in the subcortex. These findings were in line with previous studies on heritability of cortical functional connectivity and gradients^41,81^. Our findings broaden the understanding of the genetic influence on functional variations in a whole-brain perspective by extending the existing conclusions of heritability estimations of brain functions from cortical sheet to subcortex. It should be noted that the heritability of functional gradient after FDR correction in the ABCD dataset did not meet the criteria of statistical significance, which may be related to the data quality of the ABCD compared to the HCP dataset. Therefore, for the heritability analysis of the ABCD dataset, there is still a need to further verify the reliability of the *h*^2^ results.

In this study, we demonstrated that the heritability of functional gradients aligned with a unimodal-transmodal axis, which could emerge through many possible biological pathways. For instance, individual variations in cortical and subcortical structure and function may be determined in early neurodevelopment. In other words, genetics regulate the cell number, neuronal migration, and cerebral columns, and ultimately generate specific phenotypes in the early cortical progenitor cells in embryonic periods by spatial gradients of molecular transcription factors^83,84^. These processes induce variations across individuals. In this context, the projections from subcortical nuclei to the cerebral cortex may further influence cortical parcellation and facilitate the formation of cortical unimodal-transmodal axis during early embryonic stages, leading to cortical-like hierarchy in the heritability of functional gradient based on subcortico-cortical connectivity.

Brain functions are constrained by brain structures. Multiple studies have provided evidence of structure-function coupling using multimodal interpretations of the brain, including the coupling of functional connectivity and structural connectivity^85,86^, the coupling of functional gradients and T1w/T2w ratio maps^7,67^. T1w/T2w ratio could reflect multiple microstructural features including not only the myelination degree^87^, but also the iron- and water-density, cytological variations such as dendritic arborization, cell size, and cell density^88–90^. These coupling relationships between brain function and structure also exhibit spatial heterogeneity across cerebral cortex^1,91,92^, change with age^86^, and relate to cognition^93^. Additionally, the gray matter volume of the subcortex exhibits a significant correlation with the principle functional gradient, following a heterogeneous pattern in which the unimodal networks have stronger structure-function coupling^44,94^. This relationship is also reflected in estimating genetic contributions. The heritability of structure and function significantly correspond^81^, and unimodal regions account for a stronger correlation than transmodal regions^6^. However, it remains unclear how the functional organization arises from structural constraint in large-scale gradient aspect in the subcortex, and whether genetics play an important role in these constraint relationships. In this study, we observed significant spatial and genetic correlations between functional gradients and T1w/T2w ratio in young adults. Our findings presented that the relationships from structure to function are evident in the heritability analyses, suggesting that the coupling between structure and function was generally controlled by genetic architecture. Here, we used the OpenMx/umx Toolbox for bivariate heritability and genetic correlation analysis^37,95^. Consistent with these previous studies, we reported the mean and SD of the genetic correlations (rG) without multiple comparisons correction, which due to the limitations of the heritability estimation procedure of the OpenMx^37,95^. Therefore, this result needs to be further verified on different genetic correlation analysis models in the future. A previous study has demonstrated that there is a genetic and evolutionary uncoupling of structure and function in different transmodal systems in the cerebral cortex^6^. Our results are consistent with this study and extended to the human subcortex, revealing a negative spatial correlation between the structural and functional heritability. However, whether this coupling between structure and function, and their heritability, have clear causal relationships and are regulated with development, still requires further investigation.

It is important to note that our study has certain limitations that deserve attention. First, this study was conducted at the baseline time point from the ABCD study. Consequently, we did not analyze how functional gradients and their heritability changes during the longitudinal development. In addition, considering that there exist multiple differences between the HCP and ABCD datasets, we did not interpret the differences in functional gradients between children and young adults in-depth. Second, we only leveraged subcortico-cortical functional connectivity for our analyses, omitting subcortical to subcortical connectivity. Prior studies of the cerebellum have already demonstrated that cortico-cerebellar functional gradients and intra-cerebellar functional gradients are similar^96^. Thus, we focused on the cortical to subcortical functional connectivity in this study. Third, the accuracy of the conventional ACE model may be impacted by measurement errors and data quality. The ACE model assumes that there are no measurement errors in the phenotypic data, and any measurement errors would be incorporated as part of the unique environmental component. Therefore, the estimated proportion of heritability would be less precise than the actual proportion of heritability and does not pinpoint specific genetic loci. The state-of-the-art method for discovering genes involves conducting an unbiased probe of all genetic variants, like the genome-wide association study (GWAS), should be employed to quantitatively identify specific genetic loci associated with subcortical functional gradients in the future. Last, we only leveraged the T1w/T2w ratio measured by structural MRI (sMRI) to interpret subcortical structural features. Compared to sMRI, diffusion MRI (dMRI) also has the potential to capture subcortical microstructural features in neural connections and contributes to predicting functional connectivity and neurodevelopment. Structural connectivity, which constructed by dMRI, could be leveraged in the future for allowing a more in-depth investigation into the basis of the heritability of functional gradients.

In conclusion, we constructed large-scale functional gradients based on subcortico-cortical FC and assessed their heritability in both young adults and children. We discovered a similar transitional pattern in subcortical functional gradients between young adults and children and revealed a genetic contribution in subcortex function which aligned with the unimodal-transmodal axis. These findings demonstrated that topography and heritability patterns in the subcortical regions are similar to those on the cerebral cortex, suggesting a unified and finer-grained perspective to consider the cortex and subcortex as a whole. The human subcortex is a highly dense area with many small and distinct nuclei which relevant to neurodegenerative disorders^97,98^. Therefore, our findings may offer insights for studies aiming to provide evidence of atypical subcortical connectome hierarchy as a system-level substrate in a wide range of psychiatric disorders and could contribute to promoting healthy neurodevelopment.

## Methods

### Participants

Participants were from two independent datasets: the HCP^99^ and ABCD^100,101^ datasets. For the HCP dataset, subject recruitment procedures and informed consent forms were approved by the Washington University Institutional Review Board (IRB). The ABCD study uses a single IRB that acts as the IRB of record for 19 of the 21 sites, which is located at the UCSD HRPP (Human Research Protections Program). Non-reliant sites obtained local IRB approvals. The methods were performed in accordance with relevant guidelines and regulations and approved by the Beijing Institute of Technology IRB. All ethical regulations relevant to human research participants were followed. Participants, whose data have passed the initial quality control and contained the required resting-state fMRI scans, T1w images, T2w images, and kinship information, were considered for further processing. The twin zygosity status of both the HCP and ABCD participants was only determined by genotyped data. We chose 1023 participants^40^ from the HCP S1200 release as the HCP cohort, consisting of 125 MZ twin pairs and 74 DZ twin pairs, The mean age of the HCP cohort group is 28.73 years (min: 22 years, max: 37 years), with 54% being female. Meanwhile, for the ABCD participants, our selection started from the participants who remained after the selection of ABCD-BIDS Community Collection^102^. Only data that passed the Data Analysis Imaging Center quality control are included. Data were downloaded from the NIMH Data Archive. Then, additional criteria were applied for selection: (1) Remove participants whose rsfMRI, T1w, or T2w data are incomplete. (2) Remove participants whose rsfMRI data contain more than 50% of frames with FD > 0.2 mm. (3) Remove participants whose scan duration is less than 20 min. (4) Remove all the singletons and triplets. (5) Remove participants, who were labeled as twins but did not take zygosity twin testing, as well as their family members. Finally, the chosen ABCD cohort comprised 936 participants, including 108 MZ twin pairs and 124 DZ twin pairs. The mean age of this cohort is 10.06 years (min: 8.92 years; max: 11 years), with 56% being female. Additionally, to assess the potential impact of multi-scanner variability on our heritability estimation in the ABCD dataset, we created a balanced subset that exclusively included participants from specific acquisition sites. The balanced subset retained data from three specific sites: site02, site14, and site20, all equipped with Siemens Prisma MR scanners. This balanced subset comprised of 444 participants, including 91 MZ twin pairs and 109 DZ twin pairs. The mean age of this cohort is 10.21 years (min: 8.92 years; max: 11 years), with 55% being female. The selection pipeline of the ABCD cohorts is also illustrated in Supplementary Fig. 14.

### Data acquisition and preprocessing

For the HCP dataset, images were acquired on a customized 3T Siemens Skyra scanner. Resting-state fMRI runs were acquired in 2 mm isotropic voxel using multiband echo planar imaging (EPI), where each run endures 14 min and 33 s (repetition time (TR) = 720 ms, echo time (TE) = 33.1 ms). T1w and T2w images were all acquired at 0.7 mm isotropic resolution (T1w: TR = 2400 ms, TE = 2.14 ms, TI = 1000 ms, FA = 8°; T2w: TR = 3200 ms, TE = 565 ms). The HCP data had been minimally pre-processed and procedures were consistent with the original HCP pipelines^99^ (https://github.com/Washington-University/HCPpipelines). For anatomical data, T1w and T2w images were pre-processed under distortion correction, denoising, N4 bias correction, and MNI standard space registration. We leveraged skull-stripped T1w and T2w images for analyses. For resting-state fMRI, surface-based preprocessing of resting-state fMRI data included nuisance regression, temporal censoring, and spatial smoothing. All the preprocessing procedures were detailed elsewhere^99^.

For the ABCD dataset, we leveraged ABCD-BIDS Community Collection release^102^. Images in the discovery set were acquired across 21 sites with 3T scanners from three manufacturers namely Siemens, GE, and Phillips in the discovery set. In contrast, images in the balanced subset were acquired from only 3 sites with Siemens Prisma 3T MR scanner. Resting-state fMRI runs were acquired in 2.4 mm isotropic voxel using multiband EPI with slice acceleration factor 6 and resampled to 2 mm isotropic voxel. Each run lasted for 5 min (TR = 800 ms, TE = 30 ms). T1w and T2w images were all acquired at 1.0 mm isotropic resolution (T1w: TR = 2500 ms (Siemens and GE) / 6.31 ms (Philips), TE = 2.88 ms (Siemens) / 2.9 ms (Philips) / 2 ms (GE), TI = 1,060 ms, FA = 8°; T2w: TR = 3200 ms (Siemens and GE) / 2500 ms (Philips), TE = 565 ms (Siemens) / 251.6 ms (Philips) / 60 ms (GE)). Detailed scan parameters are available on the ABCD study website: (https://abcdstudy.org/images/Protocol_Imaging_Sequences.pdf). Despite the use of the original HCP pipeline preprocessing procedures listed before, resting-state fMRI scans in the ABCD dataset were further processed by DCAN BOLD Processing (DBP) (https://github.com/DCAN-Labs/dcan_bold_processing). The additional preprocessing involves applying a GLM to denoise the fMRI data and enhancing the estimation of framewise displacement by the respiratory motion filter in the DBP. Additional details of the preprocessing procedures taken and the pipeline used are publicly available (https://zenodo.org/record/2587210).

For both datasets, we did not conduct any further processing for the structural and functional data in both the HCP and ABCD datasets.

### Subcortico-cortical functional connectivity and gradients

Subcortical-cortical functional-connectivity matrices were constructed at first. The initial step involved smoothing the original pre-processed resting-state fMRI data (Gaussian filter with the FWHM = 6 mm) using the connectome workbench command *-cifti-smoothing*. We selected 1483 ROIs on the cortical sheet^46^, which was sampled in fs_LR_32k with fs_LR_900 mesh. Prior studies have already demonstrated that the connectivity across cortical sheets could be defined from a cortical region to other 1483 ROI vertices, uniformly sampled in the fs_LR32K surface space by its functional coupling^46^. Meanwhile, we kept time series for all of the subcortical voxels to generate subcortico-cortical FC. The ROI mask of the subcortex is defined as in the HCP grayordinate space. For each subject, all runs were temporally concatenated. Following the conclusion drawn by Greene and colleagues^103^, concatenation of the runs could ensure that longer scans were leveraged to achieve higher reliability for subcortical FC. Then, Pearson’s correlation between the resting-state fMRI time series at each subcortical spatial location (31,870 voxels) and the 1483 ROIs was computed. Then, individual-level functional correlation matrices underwent r-to-z transformation using the Fisher transformation. Furthermore, to obtain group-level functional gradients, functional-connectivity matrices were averaged across all individuals. As in previous works^3,11,67,92,104^, the top 10% of all connections, which encapsulate crucial information in the connectivity profile, were retained for both individualized and group-level FC matrices.

Next, to analyze the meaningful low-dimensional functional representations of spatial relationships between each two subcortical voxels in FC matrices, we applied Laplacian Eigenmap, a non-linear dimensionality reduction technique that uses the graph Laplacian of the affinity matrix to perform the embedding. Distance between each two voxels was denoted by the cosine similarity of their corresponding rows in the FC matrix. Then, utilizing the BrainSpace toolbox^45^ (https://github.com/MICA-MNI/BrainSpace), we delineated individual-level and group-level functional gradients using individualized and group-averaged FC matrices. Subsequently, each individual-level gradient was aligned with the group-level gradient using Procrustes alignment implemented in the BrainSpace toolbox^45^, scaling them onto a common embedded connectivity space.

### Network parcellation in subcortex

Employing a similar strategy as in previous studies^51,52,64^, the segmentation of the subcortical network parcellation consists of two steps. First, for both of the HCP and ABCD datasets, we segmented the cortical network parcellations in group-level respectively based on Yeo and colleagues’ study^50^. Specifically, we first defined the connectivity profile of each vertex which was uniformly sampled in fs_LR_32k with fs_LR_900 mesh, resulting in 1483 ROI vertices across the cortex. Then, cortical 1483 ROIs were clustered into 7-network parcellations, including DN, FPN, vATN, dATN, LMB, Mot, and Vis networks. Second, we segmented subcortical network parcellations based on the connection intensity of subcortico-cortical functional connectivity per row. subcortical network for each voxel could be assigned with the same network as the most related cortical ROI in the traditional winner-take-all strategy. However, the segmentation of the atlas in the subcortex would be influenced by the unstable subcortico-cortical connections due to the lower SNR in the subcortex. To mitigate these influences, we selected the top 100 of 1483 cortical ROIs that have the strongest connectivity with each voxel in the subcortex instead of the most related ROI and assigned the network of this subcortical voxel to the network accounts for the largest proportion of the 100 cortical ROIs.

#### Parcellation confidence

As in Choi and his colleagues’ study, the parcellation would be convincing with the aid of confidence map^52^. For each subcortical voxel, the confidence value was calculated in which the fraction of the top 100 correlated cortical ROIs belonging to the assigned network. In other words, assuming that for a given subcortical voxel $i$ which has already been assigned to network $j$, there exists ${n}_{j}$ of the top 100 correlated ROIs which belong to network $j$. The confidence value of the subcortical voxel $i$ is defined as ${n}_{j}/100$.

### Heritability analyses

To estimate genetic and environmental influences on the variability of functional gradients and the T1w/T2w ratio of each subcortical voxel, we leveraged Accelerated Permutation Inference for the ACE model^47^ to perform univariate genetic analyses. ACE model was utilized to estimate the proportion of the total phenotypic variation attributable to genetic factors. By combining kinship information, the ACE model automatically conducted a heritability estimation procedure, dividing the phenotypic variance into proportion results of additive genetic (A) effects, common (C), and unique (E) environmental influences on the trait. Heritability is defined as the proportion of additive genetic effects.

1 $${h}^{2}=\frac{A}{A+C+E}$$

ACE model was applied to each voxel within the entire subcortex to generate a voxel-wise heritability estimation. The ACE model was employed on FG1, FG2, and T1w/T2w ratio to assess the extent to which genetic effects influence the functional and structural phenotypes.

By leveraging the network parcellation in the subcortex, voxel-level *h*^2^ values were averaged across different functional networks to generate the mean *h*^2^ value for each network. Distribution of data points was calculated through a nonparametric statistical method called bootstrap^105^. For each iteration of the bootstrap, half of the families were randomly selected, and the heritability was recalculated using members from the chosen families. This process was repeated 1000 times to establish a statistical index.

To estimate the genetic correlations between different traits, we have also conducted bivariate genetic analyses through the umx toolbox (a user-friendly interface based on the OpenMx toolbox) function called *umxACE*^55^. In this function, bivariate ACE models were established to estimate coheritability, which is defined as the genetic covariance of standardized traits. Only voxels that have passed the LRT with *P* < 0.05 (please refer to “Statistical analyses” section) in the univariate genetic analyses of both traits were used for bivariate analyses. At each significantly heritable voxel, z-score normalization was performed on the values obtained from individual-level FG1, FG2, and T1w/T2w ratio of the participants in the HCP and ABCD datasets to obtain standardized traits. Then, the genetic correlation (rG) was calculated using both the results from univariate and bivariate genetic analyses. rG is defined as in previous studies^106^.

2 $${rG}=\frac{{h}_{{xy}}}{\sqrt{{h}_{x}^{2}{h}_{y}^{2}}}$$

Where ${h}_{{xy}}$ denotes the coheritability of trait $x$ and $y$, which is obtained from bivariate analyses. ${h}_{x}^{2}$ and ${h}_{y}^{2}$ are the heritability estimates from univariate analyses of trait $x$ and $y$, respectively.

To validate the influences of nuisance variables, a nuisance regression was implemented on the functional gradients of both datasets. The participants’ ${age},{sex},{age}\times {sex},{{age}}^{2}$ and ${{age}}^{2}\times {sex}$ were accounted for as covariates regressed by SurfStat Toolbox with a GLM before estimating heritability.

### SNR analyses

The SNR was represented by the Signal-to-Fluctuation-Noise Ratio (SFNR), which has been proven to be more important than the SNR for the fMRI data^107,108^. SFNR was calculated for each voxel separately by dividing the average signal over time by its standard deviation:

3 $${SFNR}=\frac{\frac{1}{n}\mathop{\sum }_{t=1}^{n}S\left(t\right)}{{\sigma }_{t}}$$

Here, ${\sigma }_{t}$ is the standard deviation over time for a particular voxel with an observed signal $S(t)$. SFNR was averaged over the entire brain to obtain a single value for each subject and session. Furthermore, voxel-wise SFNR maps were produced.

### Machine learning-based twin classifier

We selected the SVM classifier for training to distinguish between each two kinds of kinship relationships in the HCP dataset.

#### Definition of the distance

The between-individual variability of FG1 for every subcortical voxel *A* was defined as follows:

4 $${{Dist}}_{{Aij}}=\left|{x}_{{Ai}}-{x}_{{Aj}}\right|$$

where ${{Dist}}_{{Aij}}$ denotes the distance between *i*th and *j*th individual of *A*th voxel in subcortex, and ${x}_{{Ai}}$ and ${x}_{{Aj}}$ denote the FG1 value of the *i*th and *j*th participant, and ${{{{{\rm{|}}}}}}{{{{{\rm{\bullet }}}}}}{{{{{\rm{|}}}}}}$ denotes the absolute value of the difference. For each two individual-level FG1 maps, we calculated the distance for every single voxel in the subcortex, yielding a distance vector ($31870{voxels}* 1$). Therefore, the relationship of the input pairs was transferred into voxel-level feature differences which could be extracted for classification by the SVM.

#### SVM classification

Seven SVM classification analyses were conducted in our study, namely MZ – DZ, SIB – MZ, SIB – DZ, SIB – UNR, UNR – MZ, UNR – DZ, and UNR – Twins classifiers. All of the classifications leveraged individual-level FG1 for training and testing. The participants’ age, sex, and in-scanner head motion were accounted for as covariates regressed by SurfStat Toolbox with a GLM. The number of participants selected for each of the classifications are listed as follows: (1) MZ – DZ classification included 74 DZ twin pairs and randomly selected 74 MZ twin pairs. (2) SIB – MZ classification included 125 MZ twin pairs and randomly selected 125 SIB pairs. (3) SIB – DZ classification included 74 DZ twin pairs and randomly selected 74 SIB pairs. (4) SIB – UNR classification included 636 SIB pairs and randomly selected 636 UNR pairs. (5) UNR – MZ classification included 125 MZ twin pairs and randomly selected 125 UNR pairs. (6) UNR – DZ classification included 74 DZ pairs and 74 randomly selected UNR pairs. (7) UNR – Twins classification included 199 Twin pairs (comprised of 125 MZ pairs and 74 DZ pairs) and 199 randomly selected MZ pairs. The chosen pairs were further randomly divided into training set and testing set, keeping a ratio of 4:1. We initially employed a 5F-CV procedure to optimize the SVM parameters since they would affect the classification performance. A grid search strategy was applied to determine suitable *C* and *Gamma* values for this task. The grid search strategy found the most suitable parameters for each of the seven classifiers mentioned above. Searching for the best *C* and *Gamma* spanned both in the range [2^−5^, 2^−4.8^, …, 2^4.8^, 2^5^]. For each classification task, the SVM was trained on the training set and applied to classify the relationship of pairs in the testing set. Finally, accuracy was defined as the proportion of the correct classifications in the testing set. The mean accuracy was calculated by averaging the accuracy of 100 iterations for each classifier separately.

#### Interpreting model feature weights

To interpret the weight maps of SVM, we inverted the classification model into a forward model^57,58^. Haufe’s inversion approach yields a positive (or negative) predictive feature value (Haufe-transformed weight map) for the weight map of SVM, indicating to what extent the results of model classification attributes to the difference between FG1 at a given position. The mean Haufe-transformer weight map was obtained by averaging the absolute values of Haufe-transformed weight map of each classification run. By utilizing 7-network parcellations in the subcortex, we also averaged the absolute values across all 7-network parcellations in each Haufe-transformed weight map. Distribution of data points was calculated based on 100 times repetitions using the 7-network parcellations.

### T1w/T2w ratio

In both the HCP and ABCD datasets, individual T1w and T2w images were pre-processed through the HCP pipeline mentioned in the “Data acquisition and preprocessing” section. Individual-level T1w/T2w ratio maps were constructed by dividing the T1w image by the T2w image for each participant in the HCP and ABCD datasets. Furthermore, for comparison with functional gradient images, T1w/T2w ratio images for each individual were down-sampled to the MNI standard 2 mm voxel space using AFNI command *3dresample*. The individual T1w/T2w ratio images from the ABCD dataset underwent additional transformation through z-score normalization to mitigate intensity variability across different MR scanner types. Finally, to create a group-level T1w/T2w ratio map, we averaged individual-level T1w and T2w images in the HCP and ABCD datasets, respectively. Group-averaged T1w images were divided by group-averaged T2w images to construct a group-level T1w/T2w ratio map.

### Statistical analyses

#### Statistical analyses in network-level heritability estimations

One-way repeated analysis of variance (ANOVA) and two-tailed two-sample *t*-tests were conducted to analyze the difference between the unimodal and transmodal networks using the statistical indices established from the bootstrap computation. For each heritability estimation in the bootstrap computation, the samples of the ANOVA were obtained from the mean *h*^2^ values of the seven functional networks, and the samples of the t-test were obtained from the mean *h*^2^ values of either unimodal or transmodal regions. Data were assumed to be normal, and no gross violations of data normalcy were observed since the distribution of all data going into analyses can be observed in the relevant violin plots (Supplementary Fig. 15).

#### Significance of heritability estimations

To test the significance of our heritability estimations, we employed a conventional approach called LRT which has long been used for testing the significance of heritability in the ACE model^47^. The LRT compares the fitted model (H1: *A* > 0) to the null model (H0: *A* = 0). Specifically, the proportion of additive genetic effects was constrained to zero in a null model, and then compared with the likelihood of the fitted model to assess the significance of the heritability estimate. Twice the difference between the log-likelihoods of the null and estimated model yields a test statistic, which is asymptotically followed by a 50:50 mixture of chi-square distributions, i.e., ${\chi }_{0}^{2}/2+{\chi }_{1}^{2}/2$.

#### Significance of classification performance

We conducted a permutation test to evaluate whether the classification performance was significantly higher than expected by chance^109^. The individuals’ actual relationship labels were randomly scrambled 1000 times, with each iteration involving the training and classification procedures. Finally, a null distribution was established, and the observed classification accuracy was compared with the null distribution to obtain the ranking value. The *P*-value was defined as the proportion of permutation accuracies that are higher than the actual accuracy of the model.

#### Spatial randomization testing

To evaluate the significance of the spatial correlation between the maps of functional gradients and T1w/T2w ratio, as well as their heritability analyses, we employed the spatial permutation framework named the Moran Spectrum Randomization^45^. Adopted from the ecology literature, the Moran Spectrum Randomization could be used to generate random variables with identical or similar spatial autocorrelation (in terms of Moran’s I correlation coefficient), which constitute a more conservative null distribution than randomly shuffling the subcortical voxel locations^110^. Thus, the null distribution generated by the Moran Spectrum Randomization preserved the original spatial characteristics of the feature map to a greater extent. This approach requires building a spatial weight matrix defining the relationships between the different locations. We therefore computed the spatial distance between each two voxels in the subcortex using Euclidean distance under the MNI152 coordinates. The significance level *P*_moran_ was calculated as the proportion of times that the observed correlation was lower than the null distributions which were generated by the Moran Spectrum Randomization strategy.

### Reporting summary

Further information on research design is available in the Nature Portfolio Reporting Summary linked to this article.

### Supplementary information

Supplementary Information

Description of Additional Supplementary Materials

Supplementary Data 1

Reporting Summary

## Data Availability

The raw and pre-processed data are available from the Human Connectome Project (https://www.humanconnectome.org/study/hcp-young-adult/document/1200-subjects-data-release) and Adolescent Brain Cognitive Development Study (https://abcdstudy.org/scientists/data-sharing/). The chosen participants’ IDs and their kinship information of both the HCP and ABCD datasets are listed at our GitHub page (https://github.com/BIT-YangLab/Subcortical_Heritability). Numerical source data generated in this study are included in Supplementary Data 1.

## Code availability

The code for analyzing the data could be found at our GitHub page (https://github.com/BIT-YangLab/Subcortical_Heritability). The BrainSpace Toolbox for constructing functional gradients is available at https://github.com/MICA-MNI/BrainSpace. The Moran Spectrum Randomization used in generating null data is also implemented in the BrainSpace Toolbox. In the heritability estimations, the APACE Toolbox for univariate genetic analyses (https://github.com/NISOx-BDI/APACE) and the umx Toolbox for bivariate genetic analyses (https://github.com/tbates/umx) are both publicly available. Surface visualization of the cerebellum was generated with SUIT toolbox^111^ (https://www.diedrichsenlab.org/imaging/suit.htm). Volume visualization of subcortex in 3-D scatters is publicly available (https://github.com/wd-veloce/plot_fig_subcortex). The support vector machine was implemented in toolbox LIBSVM. The nuisance regression leveraged the SurfStat Toolbox (https://math.mcgill.ca/keith/surfstat/).
