# Supplementary material for: Heritability of functional gradients in the human subcortico-cortical connectivity
Source: Commun Biol. 2024 Jul 12;7:854. doi: 10.1038/s42003-024-06551-5 (PMC11245549; doi:10.1038/s42003-024-06551-5)
Supplement: Supplementary file 1 — Supplementary Information [file 42003_2024_6551_MOESM1_ESM.pdf]

# Supplementary Information

## Supplementary Figure 1

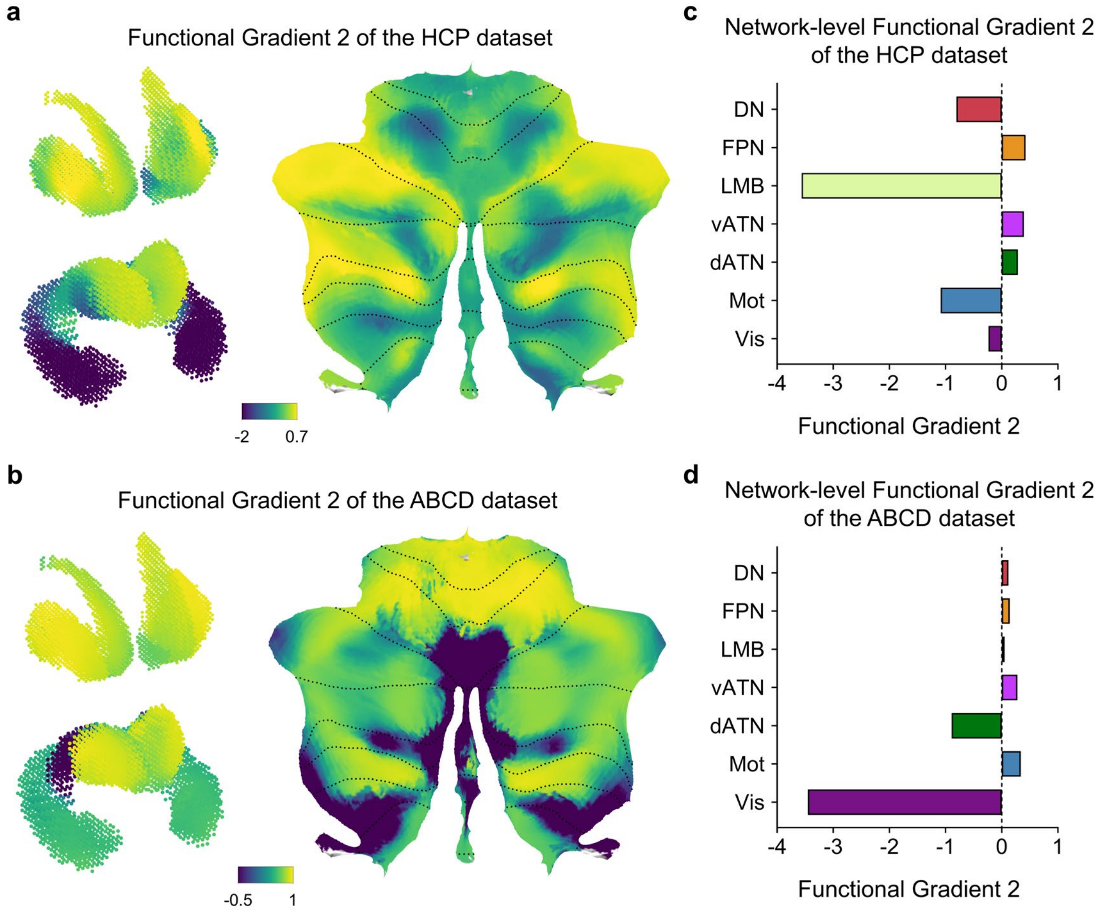


**Supplementary Fig. 1 Group-level FG2 of the subcortex for the HCP and ABCD datasets. a-b** Group-level FG2 in striatum, hippocampus, thalamus and cerebellum of the HCP and ABCD datasets, respectively. **c-d** Unlike FG1, network-level distribution of the FG2 values is different between the HCP and ABCD datasets. The definition of the 7-network parcellation in subcortex could be found in Fig. 1c. Please see Methods for further details. DN, default mode network; FPN, frontoparietal network; LMB, limbic network; vATN, ventral attention network; dATN, dorsal attention network; Mot, somatomotor network; Vis, visual network.

## Supplementary Figure 2

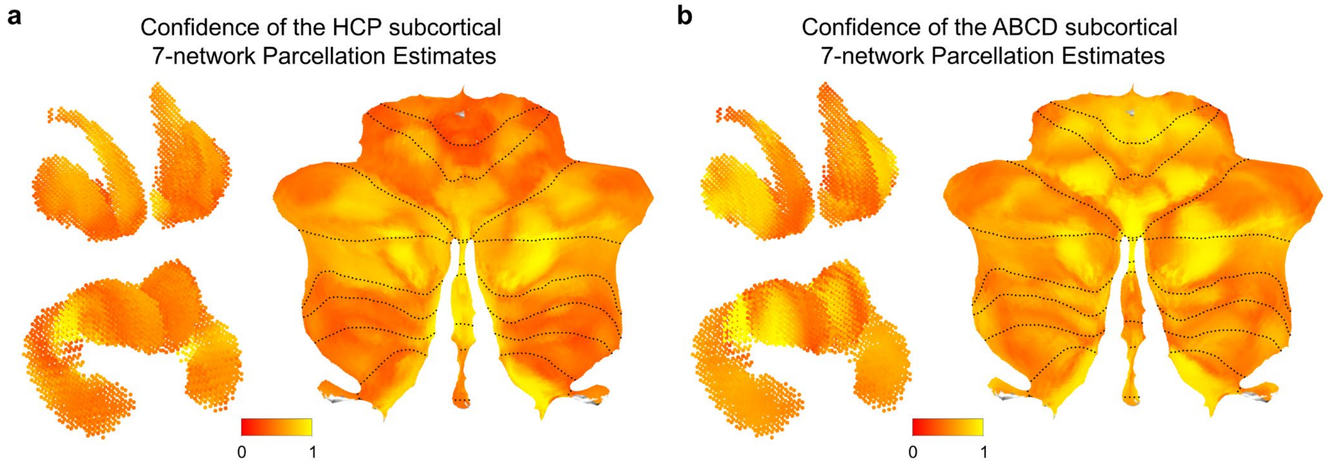


**Supplementary Fig. 2 Confidence maps of the HCP and ABCD subcortical parcellation. a-b** Confidence of the subcortical parcellation estimates of the HCP and ABCD datasets, respectively. The values at each voxel indicates the fraction of the top 100 correlated cortical vertices belonging to the assigned network. Across subcortical regions, 60.24% voxels in the HCP dataset and 74.79% voxels in the ABCD dataset achieve a confidence value exceeding 0.5.

## Supplementary Figure 3

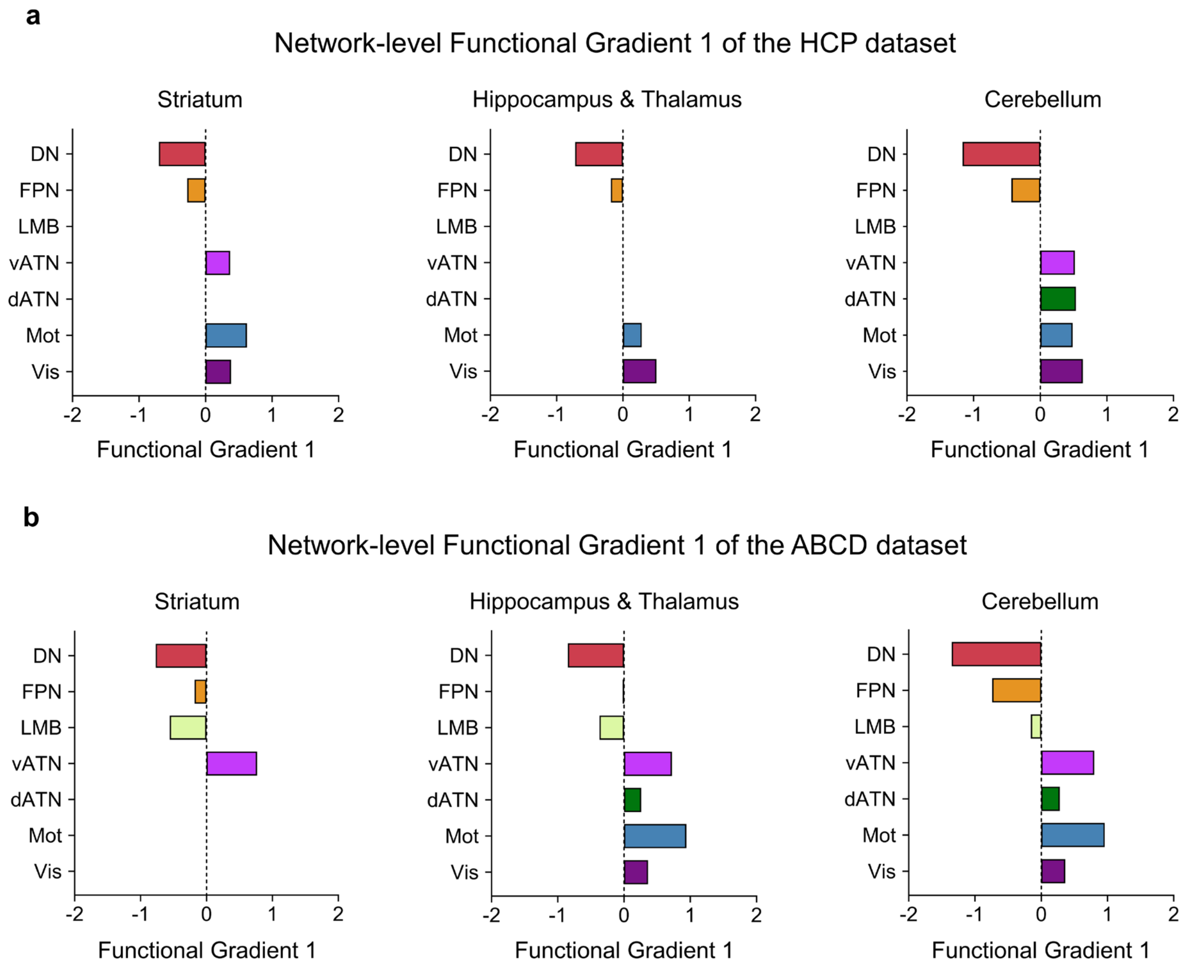


**Supplementary Fig. 3 The network-level FG1 in different subcortical nuclei also exhibits similar transitions from transmodal networks to unimodal networks in both the HCP and ABCD datasets. a-b** The network-level FG1 in each subcortical nuclei, including striatum, hippocampus, thalamus and cerebellum in young adults (HCP) and children (ABCD). FG1 in subcortical nuclei also shows similar transitions as in the whole subcortex.

## Supplementary Figure 4

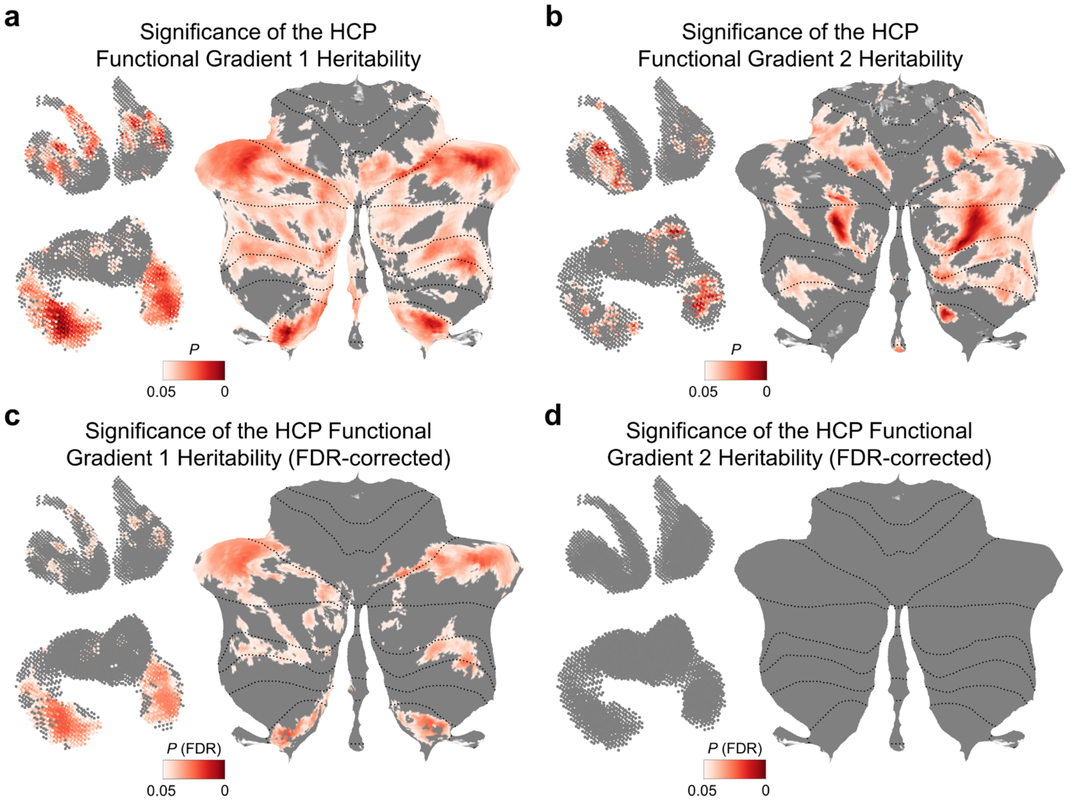


**Supplementary Fig. 4 The heritability of both the FG1 and FG2 in transmodal networks were significantly controlled by genes in the HCP dataset. a-b** The significance is tested by LRT statistics of the heritability of FG1 and FG2 in the HCP dataset. Most positions where *P*-values less than 0.05 locate in transmodal networks. **c-d** Significance level of the heritability of FG1 and FG2 in the HCP dataset was reported with the multiple comparison corrected P-values. Red and white colour denotes $P(\text{FDR}) < 0.05$ and gray colour denotes $P(\text{FDR}) > 0.05$.

## Supplementary Figure 5

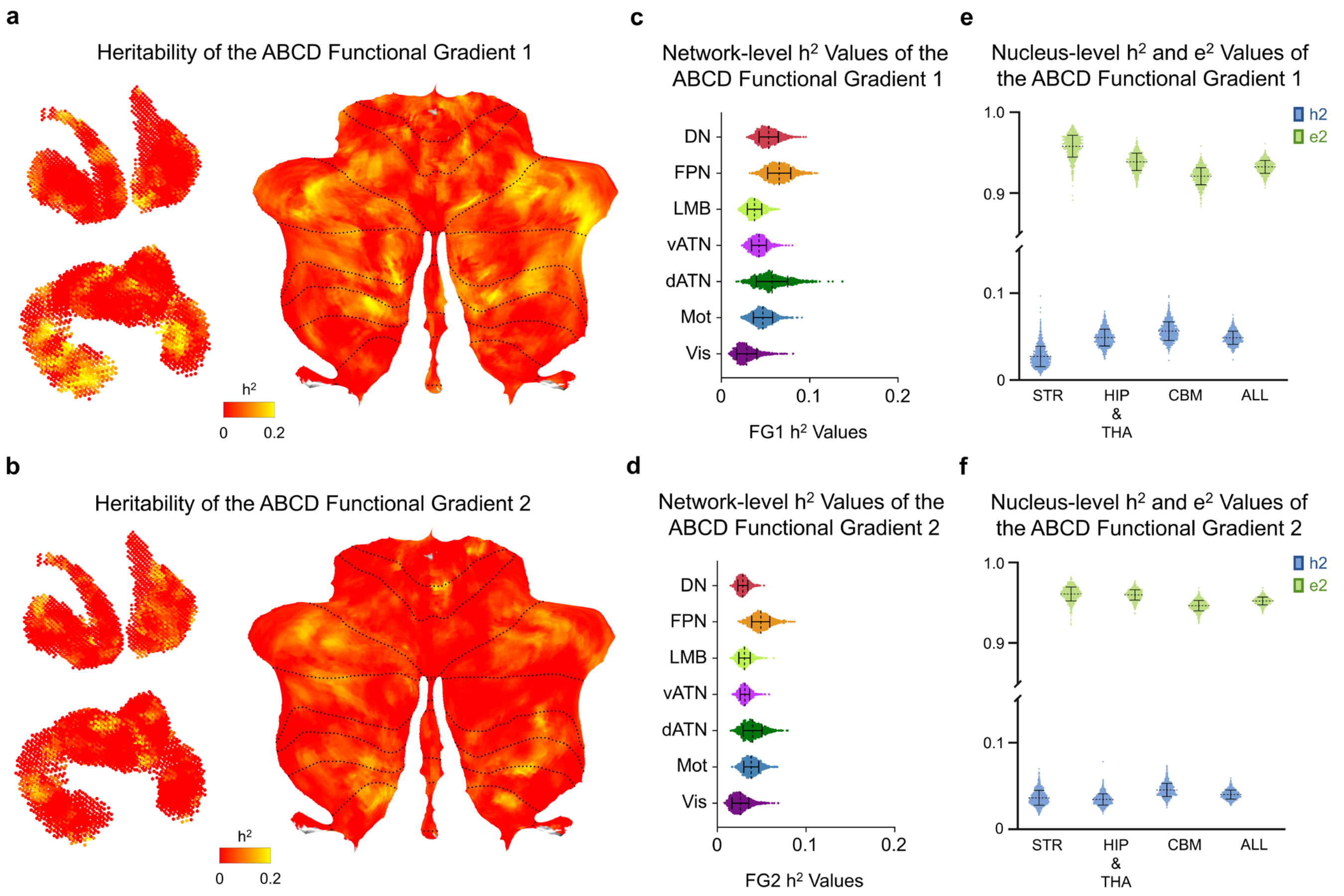


**Supplementary Fig. 5 Heritability of the ABCD FG1 and FG2 in the subcortex. a** Heritability maps of FG1 in striatum, thalamus, hippocampus, and cerebellum in the ABCD dataset. **b** Heritability maps of FG2 in the subcortex in the ABCD dataset. **c-d** The heritability of both FG1 and FG2 in the ABCD dataset represented by $h^2$ index exhibit a decrease pattern from transmodal to unimodal networks. Mean heritability estimation values are the highest in frontoparietal network in both the ABCD FG1 and FG2. Error bar reflects standard deviations obtained by bootstrap strategy. **e-f** The additive genetic and unique environmental contribution to the FG1 and FG2 in the subcortex of the ABCD dataset. Unlike the results in the HCP dataset, genetic contributions on each subarea are more closely approximated in both the FG1 and FG2 in the ABCD dataset. ALL, whole subcortex; STR, striatum; HIP & THA, hippocampus and thalamus; CBM, cerebellum.

## Supplementary Figure 6

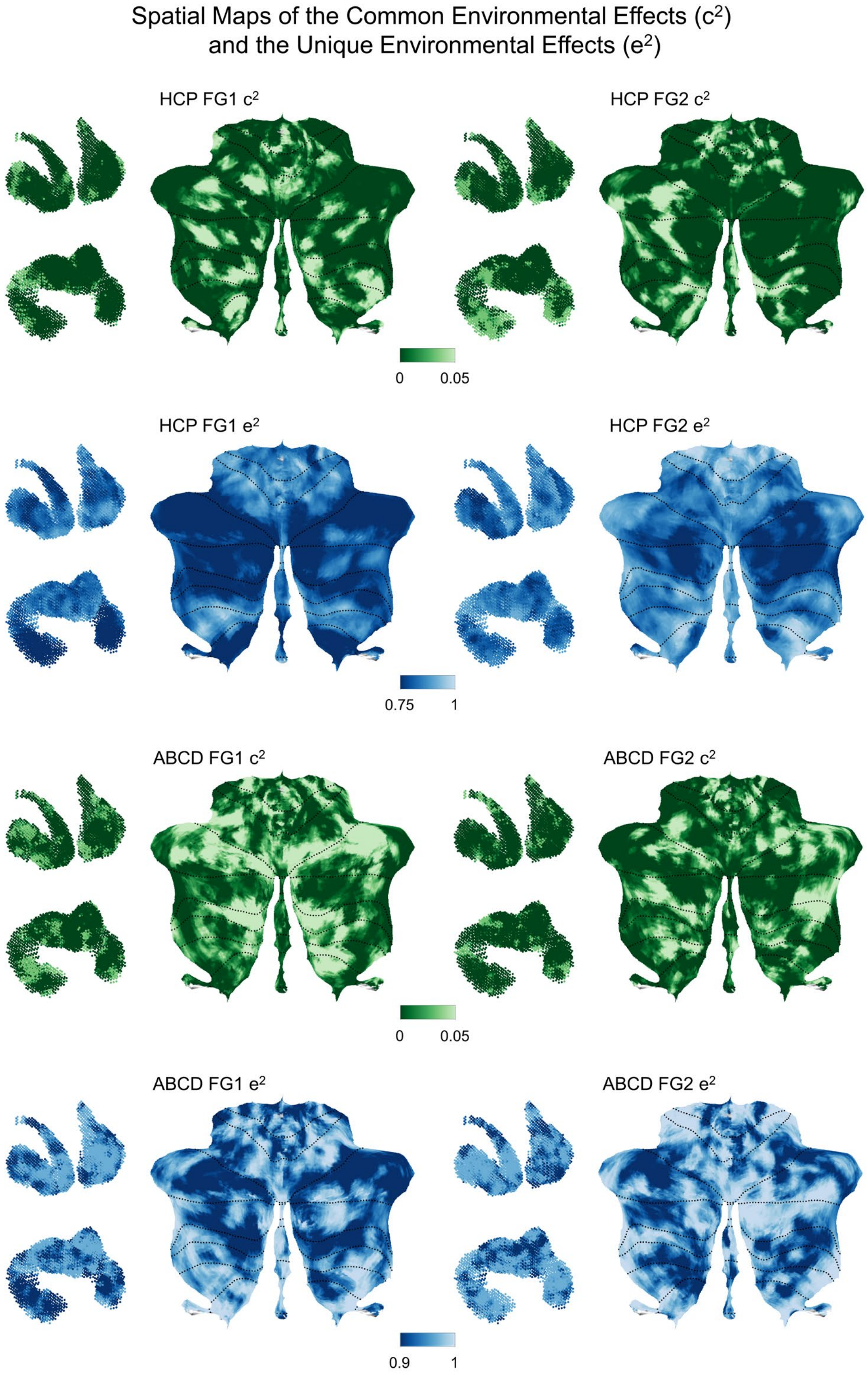


**Supplementary Fig. 6 Spatial maps of the common environmental effects ($c^2$) and the unique environmental effect ($e^2$).** The spatial maps of the common environmental effects ($c^2$) and the unique environmental effect ($e^2$) estimations of the FG1 and FG2 in both the HCP and ABCD datasets. The Spatial maps of $e^2$ exhibit a similar but reverse pattern to the heritability estimations, while those of $c^2$ are relatively sparse and random for both the HCP and ABCD datasets.

## Supplementary Figure 7

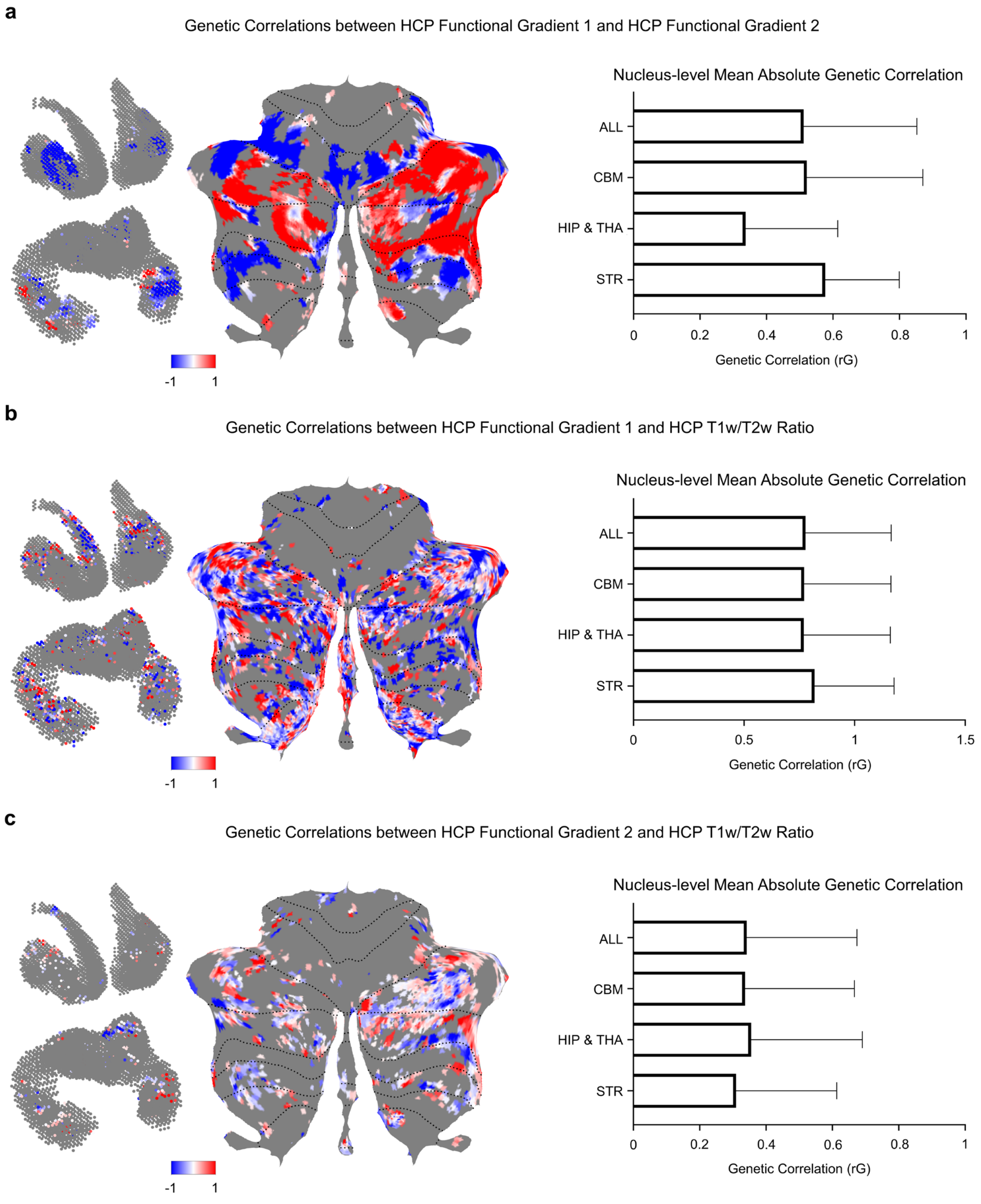


**Supplementary Fig. 7 Strong genetic correlations existed among the FG1, FG2 and T1w/T2w ratio in the HCP dataset**. **a** The genetic correlation between FG1 and FG2 in the HCP dataset ($Mean_{|rG|} = 0.512$, $SD_{|rG|} = 0.341$). **b** The genetic correlation between FG1 and T1w/T2w ratio in the HCP dataset ($Mean_{|rG|} = 0.778$, $SD_{|rG|} = 0.387$). **c** The genetic correlation between FG2 and T1w/T2w ratio in the HCP dataset ($Mean_{|rG|} = 0.342$, $SD_{|rG|} = 0.332$). Gray regions indicate that the voxels did not passed the LRT in univariate genetic analysis of both traits and were not used to calculate genetic correlations. Error bar reflects standard deviations. ALL, whole subcortex; STR, striatum; HIP & THA, hippocampus and thalamus; CBM, cerebellum.

## Supplementary Figure 8

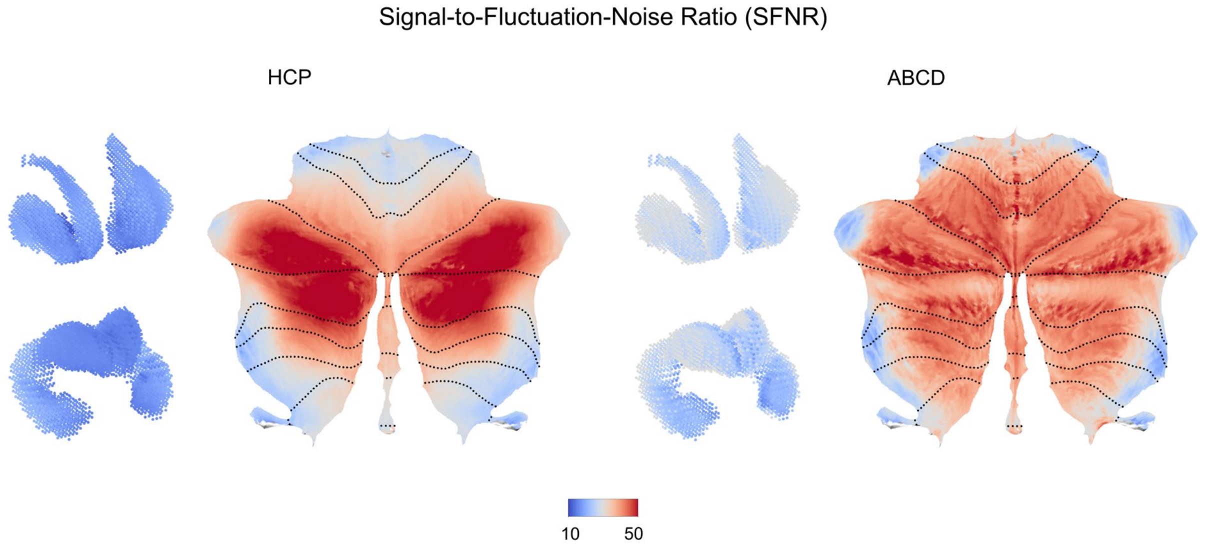


**Supplementary Fig. 8 Signal-to-Fluctuation-Noise Ratio of the striatum, hippocampus, thalamus and cerebellum.** Although the heritability results would be affected by the data quality of the fMRI scans, the heritability estimations should not be only interpreted as the reflection of SNR. Specifically, the SFNR correlates with the heritability in the HCP dataset (SFNR - FG1 $h^2$: Spearman's $r = 0.483$, $P_{moran} < 0.001$; SFNR – FG2 $h^2$: Spearman's $r = 0.498$, $P_{moran} < 0.01$). However, there are no significant associations between the SFNR and the heritability in the ABCD dataset (SFNR - FG1 $h^2$: Spearman's $r = -0.007$, $P_{moran} = 0.4$; SFNR – FG2 $h^2$: Spearman's $r = 0.044$, $P_{moran} = 0.117$).

## Supplementary Figure 9

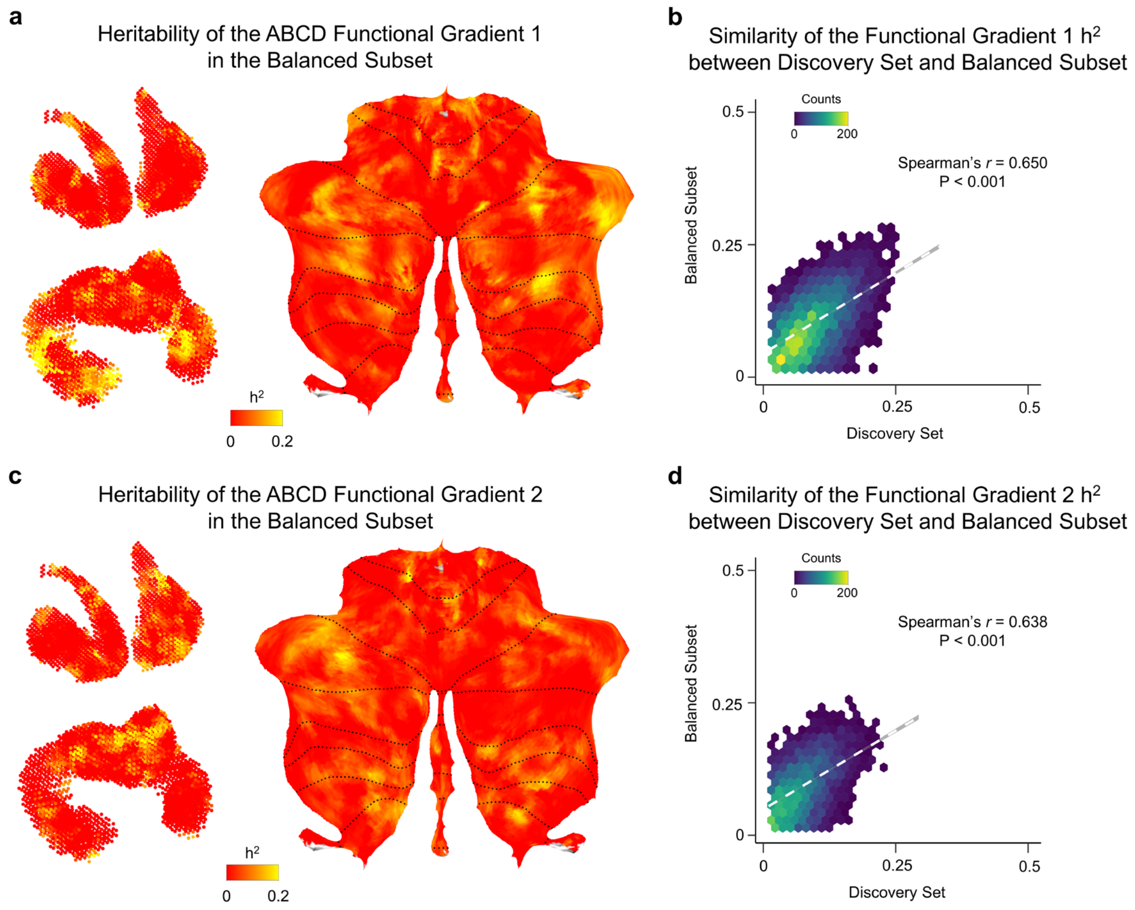


**Supplementary Fig. 9 The heritability of the FG1 and FG2 in the balanced subset are similar with those in the discovery set. a** Heritability maps of FG1 in striatum, thalamus, hippocampus, and cerebellum in the balanced subset. **b** The heritability of FG1 are significantly similar between the discovery set and balanced subset (Spearman's $r = 0.650$, $P_{\text{moran}} < 0.001$). **c** Heritability maps of FG2 in striatum, thalamus, hippocampus, and cerebellum in the balanced subset. **d** The heritability of FG2 are also similar between the discovery set and balanced subset (Spearman's $r = 0.638$, $P_{\text{moran}} < 0.001$).

## Supplementary Figure 10

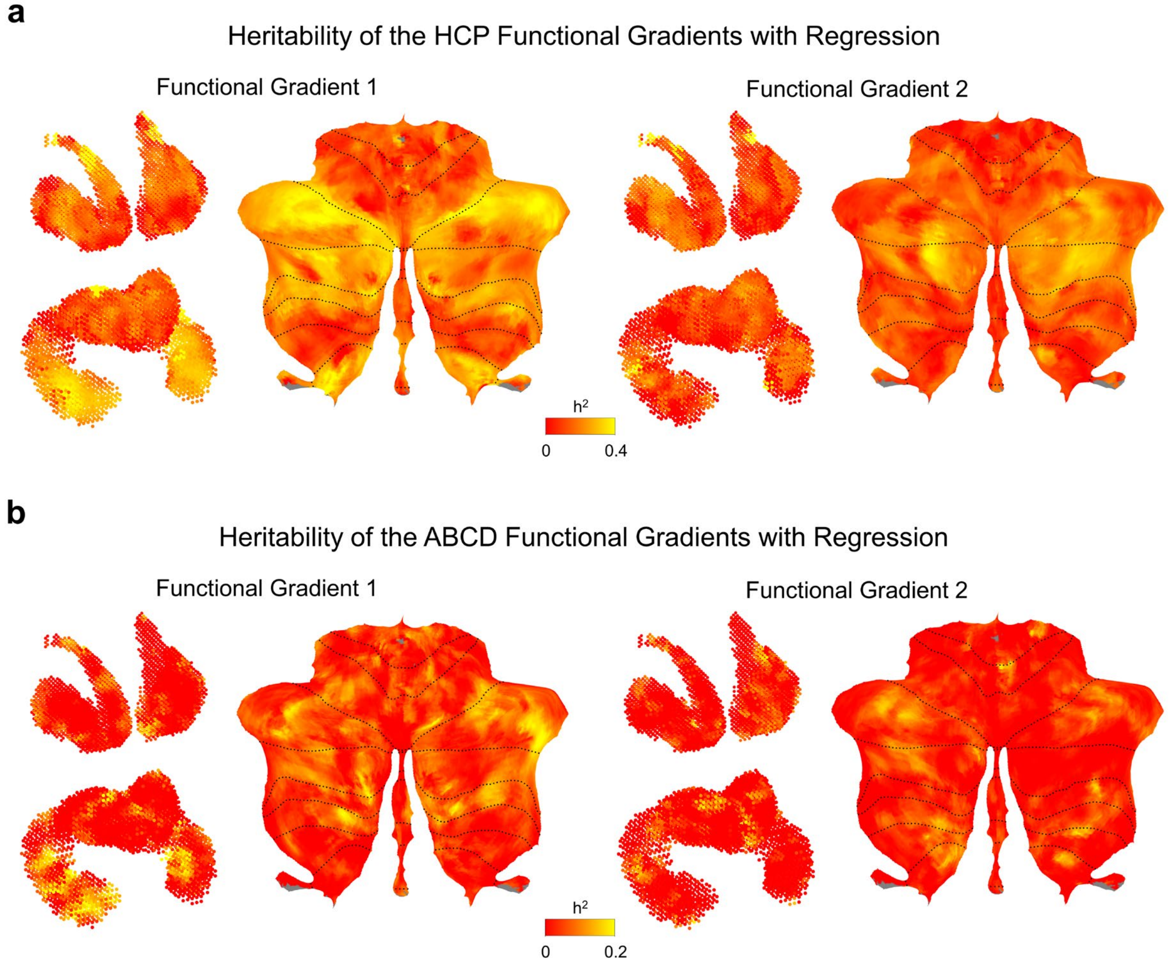


**Supplementary Fig. 10 The heritability of the FG1 and FG2 in both the HCP and ABCD datasets after nuisance regression** The participants' $age, sex, age \times sex, age^2$ and $age^2 \times sex$ were accounted for as covariates regressed by SurfStat Toolbox with a general linear model (GLM) before estimating heritability. It is highly similar between the results with and without nuisance regression (HCP FG1: Spearman's $r = 0.957$; HCP FG2: Spearman's $r = 0.965$; ABCD FG1: Spearman's $r = 0.981$; ABCD FG2: Spearman's $r = 0.975$).

## Supplementary Figure 11

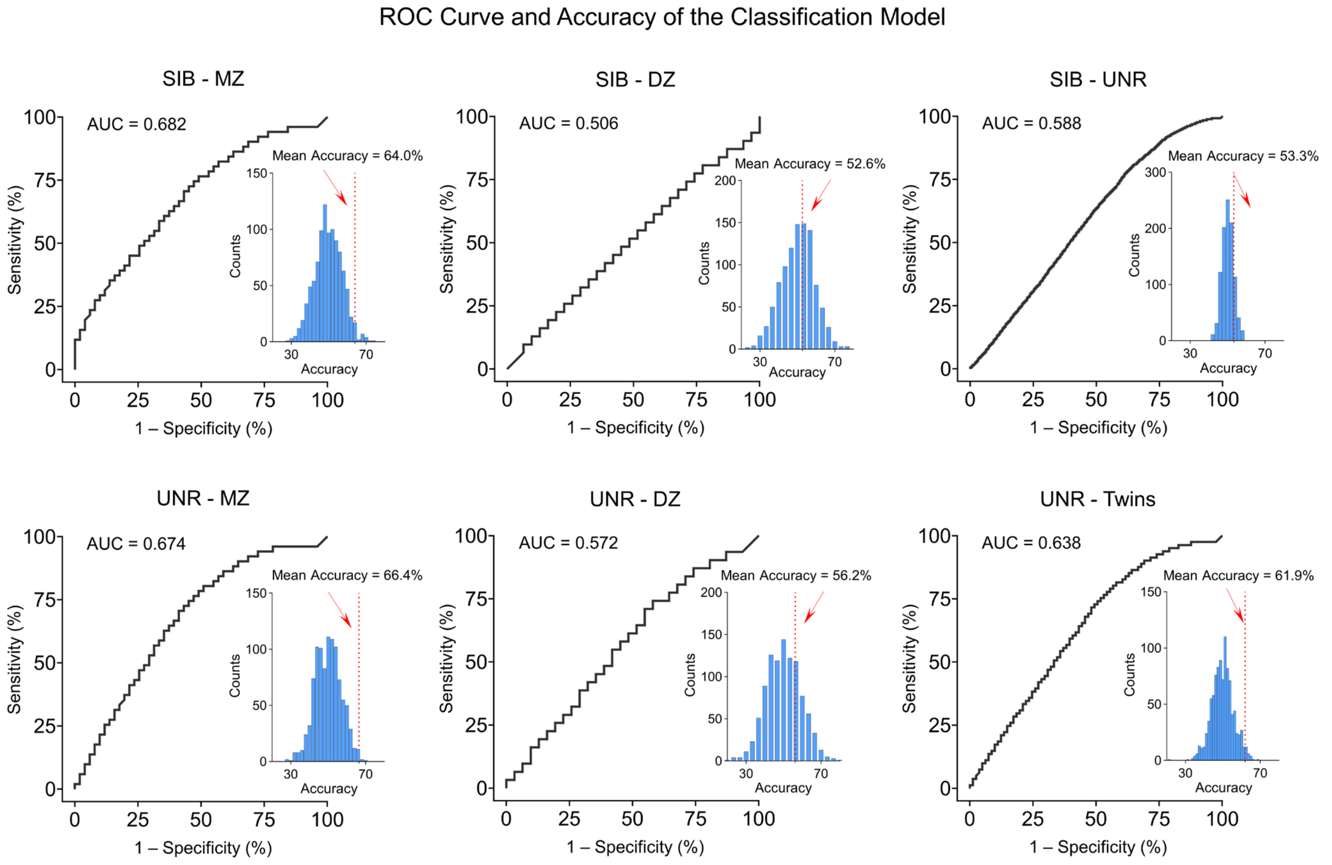


**Supplementary Fig. 11 ROC Curve and accuracy of the classification model.** SVM classifiers involving the siblings and unrelated individuals, namely (1) SIB – MZ classifier (ACC = 64.0%, AUC = 0.682), (2) SIB – DZ classifier (ACC = 52.6%, AUC = 0.506), (3) SIB – UNR classifier (ACC = 53.3%, AUC = 0.588), (4) UNR – MZ classifier (ACC = 66.4%, AUC = 0.674), (5) UNR – DZ classifier (ACC = 56.2%, AUC = 0.572) and (6) UNR – Twins (MZ and DZ) classifier (ACC = 61.9%, AUC = 0.638). The results show that the accuracies of these classifiers are relatively poorer than the MZ – DZ classifier. ACC, accuracy; AUC, area under the curve.

## Supplementary Figure 12

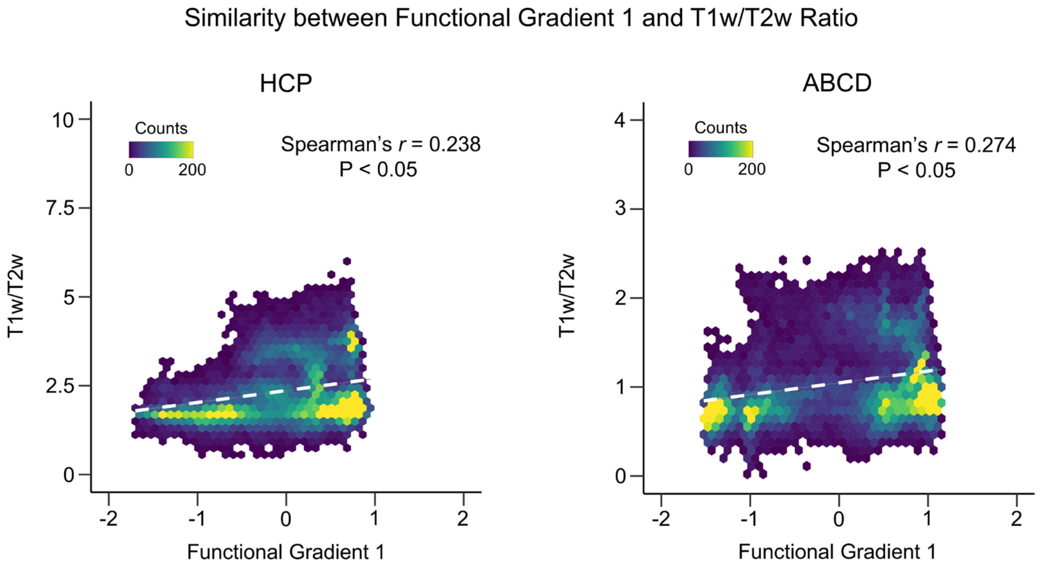


**Supplementary Fig. 12 Group-level principle functional gradients exhibit significant correlation with the T1w/T2w ratio maps.** The spatial maps of the group-level principle functional gradients significantly associated with T1w/T2w ratio in both the HCP (Spearman's $r = 0.238$, $P < 0.05$) and the ABCD (Spearman's $r = 0.274$, $P < 0.05$) datasets.

## Supplementary Figure 13

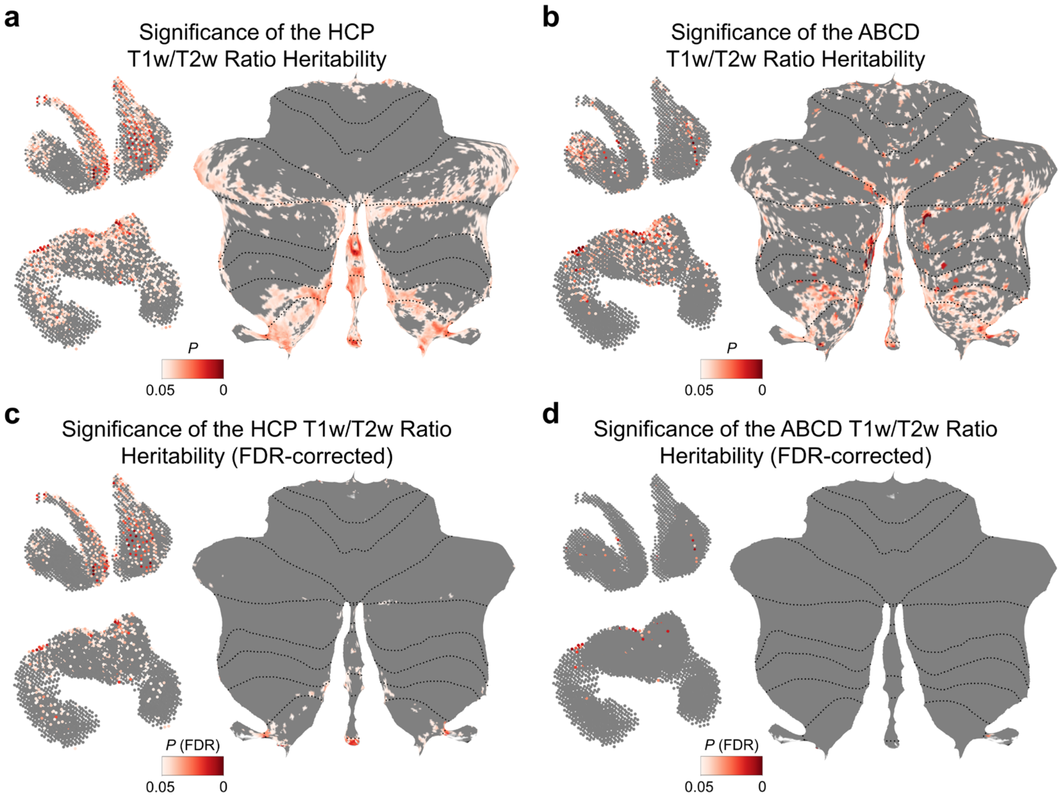


**Supplementary Fig. 13 The heritability of T1w/T2w ratio maps in transmodal networks were significantly controlled by genes in both the HCP and ABCD datasets. a-b** The significance is tested by LRT statistics of the heritability of T1w/T2w ratio in both the HCP and ABCD datasets. **c-d** Significance level of the heritability of T1w/T2w ratio in both the HCP and ABCD datasets was reported with the multiple comparison corrected $P$-values. Most positions where $P$(FDR) < 0.05 also locate in transmodal networks. Red and white colour denotes $P$(FDR) < 0.05 and gray colour denotes $P$(FDR) > 0.05.

## Supplementary Figure 14

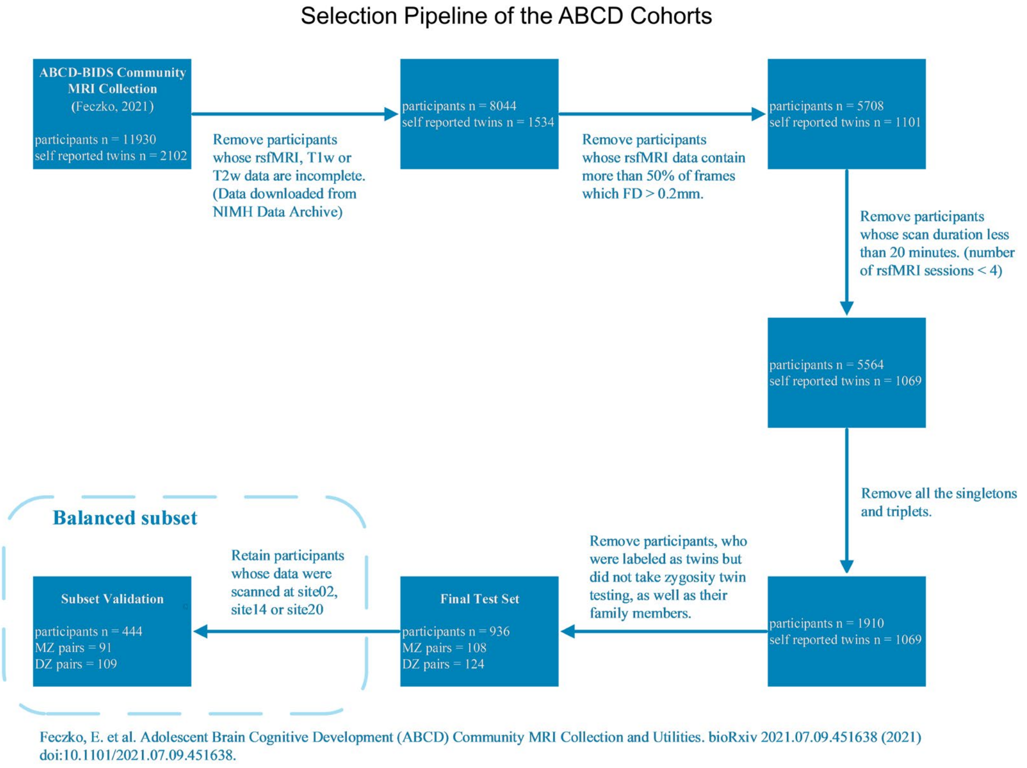


**Supplementary Fig. 14 Pipeline of the participant selection in the ABCD dataset.** The pipeline illustrated the selection of ABCD cohorts. Text on the arrows illustrate the rules to select the cohorts. Each blue rectangle box showing the number of cohorts remaining after each selection steps. The overall pipeline is demonstrated at *Method – Participants* section.

## Supplementary Figure 15

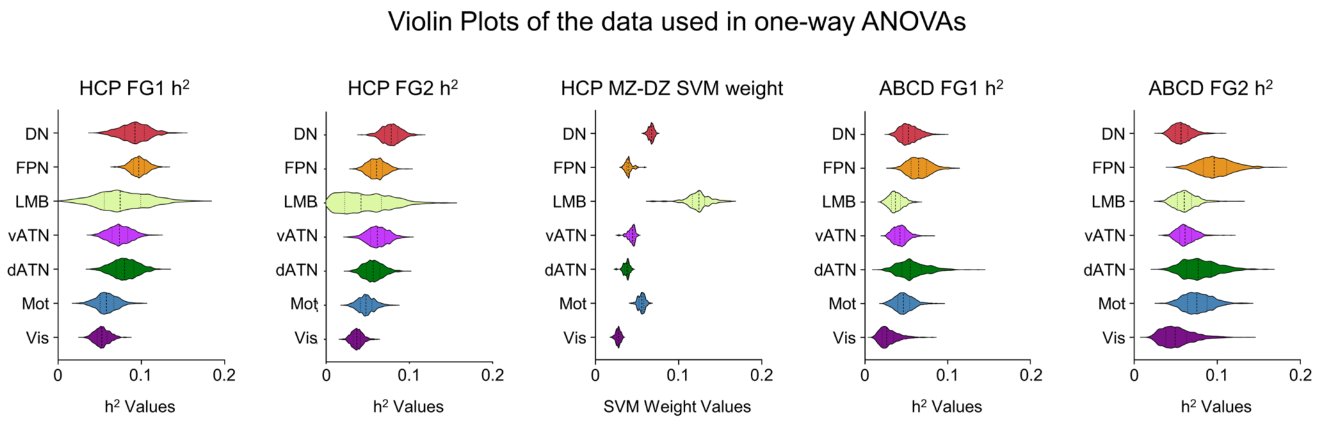


**Supplementary Fig. 15 Violin plots of the data used in one-way ANOVAs** Data were assumed to be normal and no gross violations of data normalcy were observed.
