# Supplementary material for: Heritability of functional gradients in the human subcortico-cortical connectivity
Source: Commun Biol. 2024 Jul 12;7:854. doi: 10.1038/s42003-024-06551-5 (PMC11245549; doi:10.1038/s42003-024-06551-5)
Supplement: Supplementary file 2 — Description of Additional Supplementary Materials [file 42003_2024_6551_MOESM2_ESM.pdf]

Description of Additional Supplementary Files

File name: Supplementary Data 1

Description: Numerical source data generated in this study
