# Supplementary material for: Heritability of functional gradients in the human subcortico-cortical connectivity
Source: Commun Biol. 2024 Jul 12;7:854. doi: 10.1038/s42003-024-06551-5 (PMC11245549; doi:10.1038/s42003-024-06551-5)
Supplement: Supplementary file 4 — Reporting Summary [file 42003_2024_6551_MOESM4_ESM.pdf]

nature portfolio

Corresponding author(s): Guoyuan Yang

Last updated by author(s): Dec 5, 2023

Reporting Summary

Nature Portfolio wishes to improve the reproducibility of the work that we publish. This form provides structure for consistency and transparency in reporting. For further information on Nature Portfolio policies, see our Editorial Policies and the Editorial Policy Checklist.

Statistics

For all statistical analyses, confirm that the following items are present in the figure legend, table legend, main text, or Methods section.

n/a	Confirmed	
☐	☒	The exact sample size (*n*) for each experimental group/condition, given as a discrete number and unit of measurement
☐	☒	A statement on whether measurements were taken from distinct samples or whether the same sample was measured repeatedly
☐	☒	The statistical test(s) used AND whether they are one- or two-sided<br>*Only common tests should be described solely by name; describe more complex techniques in the Methods section.*
☒	☐	A description of all covariates tested
☐	☒	A description of any assumptions or corrections, such as tests of normality and adjustment for multiple comparisons
☐	☒	A full description of the statistical parameters including central tendency (e.g. means) or other basic estimates (e.g. regression coefficient) AND variation (e.g. standard deviation) or associated estimates of uncertainty (e.g. confidence intervals)
☐	☒	For null hypothesis testing, the test statistic (e.g. *F*, *t*, *r*) with confidence intervals, effect sizes, degrees of freedom and *P* value noted<br>*Give P values as exact values whenever suitable.*
☒	☐	For Bayesian analysis, information on the choice of priors and Markov chain Monte Carlo settings
☒	☐	For hierarchical and complex designs, identification of the appropriate level for tests and full reporting of outcomes
☐	☒	Estimates of effect sizes (e.g. Cohen's *d*, Pearson's *r*), indicating how they were calculated

Our web collection on statistics for biologists contains articles on many of the points above.

Software and code

Policy information about availability of computer code

Data collection	No software was used for data collection.
Data analysis	Custom scripts were written using Bash Shell Scripting (version 3.1.11). Software used in the data analyses including HCP pipeline v4.0.0 (https://github.com/Washington-University/HCPpipelines/releases/tag/v4.0.0), FSL 6.0.3 (https://fsl.fmrib.ox.ac.uk/), FreeSurfer 6.0 (https://surfer.nmr.mgh.harvard.edu), WorkBench (https://www.humanconnectome.org/software/workbench-command), AFNI (https://afni.nimh.nih.gov/), LIBSVM (https://github.com/cjlin1/libsvm), BrainSpace Toolbox (https://github.com/MICA-MNI/brainspace), APACE (https://github.com/NISOx-BDI/APACE), SUIT (https://github.com/jdiedrichsen/suit), plot_fig_subcortex (https://github.com/wd-veloce/plot_fig_subcortex), MATLAB (https://www.mathworks.com/products/matlab.html) and R-Studio (https://www.rstudio.com/). All the codes and atlases used in this study are available at the GitHub repository (https://github.com/BIT-YangLab/Subcortical_Heritability).

For manuscripts utilizing custom algorithms or software that are central to the research but not yet described in published literature, software must be made available to editors and reviewers. We strongly encourage code deposition in a community repository (e.g. GitHub). See the Nature Portfolio guidelines for submitting code & software for further information.

Data

Policy information about availability of data

All manuscripts must include a data availability statement. This statement should provide the following information, where applicable:

- Accession codes, unique identifiers, or web links for publicly available datasets
- A description of any restrictions on data availability
- For clinical datasets or third party data, please ensure that the statement adheres to our policy

The Human Connectome Project (HCP) dataset used in this study is publicly available at https://dbhumanconnectome.org/. The Adolescent Brain Cognitive Development (ABCD) Study dataset including in this study is publicly available through the NIMH Data Archive (NDA) on the ABCD study data sharing webpage: https://abcdstudy.org/scientists_data_sharing.html. Instructions on how to create an NDA study are available at https://nda.nih.gov/training/modules/study.html).

Research involving human participants, their data, or biological material

Policy information about studies with human participants or human data. See also policy information about sex, gender (identity/presentation), and sexual orientation and race, ethnicity and racism.

Reporting on sex and gender	See below.
Reporting on race, ethnicity, or other socially relevant groupings	This study did not address the use of race, ethnicity, or other socially relevant groupings data.
Population characteristics	For the Human Connectome Project (HCP) dataset, we chose 1023 participants from the HCP S1200 release as the HCP cohort, consisting of 125 monozygotic (MZ) twin pairs and 74 dizygotic (DZ) twin pairs, The mean age of the HCP cohort group is 28.73 years (min: 22 years, max: 37 years), with 54% being female.<br><br>For the Adolescent Brain Cognitive Development (ABCD) Study participants, the chosen ABCD cohort comprised 936 participants, including 108 MZ twin pairs and 124 DZ twin pairs. The mean age of this cohort is 10.06 years (min: 8.92 years; max: 11 years), with 56% being female.
Recruitment	Human Connectome Project:<br>A sample of 1023 healthy participants was drawn from the HCP dataset, as publicly provided by the HCP S1200 subjects data release.<br><br>Adolescent Brain Cognitive Development Study:<br>A sample of 936 healthy participants was drawn from the ABCD dataset, as publicly provided through the NIMH Data Archive (NDA) is available on the ABCD study data sharing webpage.
Ethics oversight	Informed consent was obtained from all Human Connectome Project participants, and the procedures were approved by the Washington University Institutional Review Board (IRB). The Adolescent Brain Cognitive Development Study uses a single IRB that acts as the IRB of record for 19 of the 21 sites. This single IRB is located at the UCSD HRPP (Human Research Protections Program).

Note that full information on the approval of the study protocol must also be provided in the manuscript.

Field-specific reporting

Please select the one below that is the best fit for your research. If you are not sure, read the appropriate sections before making your selection.

☒ Life sciences ☐ Behavioural & social sciences ☐ Ecological, evolutionary & environmental sciences

For a reference copy of the document with all sections, see nature.com/documents/nr-reporting-summary-flat.pdf

Life sciences study design

All studies must disclose on these points even when the disclosure is negative.

Sample size	For both the Human Connectome Project (HCP) and Adolescent Brain Cognitive Development (ABCD) Study datasets, we all employed strict data quality control and ended up with a total of remaining data, including 1023 participants from the HCP S1200 release and 936 participants for the ABCD Study. No additional statistic analysis was performed for the choosing sample size criteria.
Data exclusions	For the HCP dataset, participants whose data have passed the initial quality control and contained the required resting-state fMRI scans, T1w images, T2w images and kinship information, were considered for further processing. The twin zygosity status of both the HCP and ABCD Study participants was only determined by genotyped data. We chose 1023 participants from the HCP S1200 release as the HCP cohort.<br><br>For the ABCD Study participants, additional criteria were applied for selection: (1) Exclusion of participants with fewer than 3 resting-state fMRI sessions or concatenated sessions containing more than 50% of frames with FD > 0.3mm. (2) Removal of participants without familial

	relatedness. (3) Exclusion of all triplet families to simplify computations. (4) Exclusion of participants labelled as twins but lacking genotyped data. Finally, the chosen ABCD cohort comprised 936 participants.
Replication	To assess the potential impact of multi-scanner variability on our heritability estimation in the ABCD dataset, we created a validation set that exclusively included participants from specific acquisition sites. The validation set retained data from three specific sites: site02, site14, and site20, all equipped with Siemens Prisma MR scanners. This validation set comprised of 444 participants, including 91 MZ twin pairs and 109 DZ twin pairs. The mean age of this cohort is 10.21 years (min: 8.92 years; max: 11 years), with 55% being female.
Randomization	Non-applicable: there was no randomization applied on this study, as all participants were healthy individuals.
Blinding	All subjects examined were healthy individuals. Therefore, blinding was not relevant in our study.

Reporting for specific materials, systems and methods

We require information from authors about some types of materials, experimental systems and methods used in many studies. Here, indicate whether each material, system or method listed is relevant to your study. If you are not sure if a list item applies to your research, read the appropriate section before selecting a response.

Materials & experimental systems

n/a	Involved in the study
☒	☐ Antibodies
☒	☐ Eukaryotic cell lines
☒	☐ Palaeontology and archaeology
☒	☐ Animals and other organisms
☒	☐ Clinical data
☒	☐ Dual use research of concern
☒	☐ Plants

Methods

n/a	Involved in the study
☒	☐ ChIP-seq
☒	☐ Flow cytometry
☐	☒ MRI-based neuroimaging

Plants

Seed stocks	*Report on the source of all seed stocks or other plant material used. If applicable, state the seed stock centre and catalogue number. If plant specimens were collected from the field, describe the collection location, date and sampling procedures.*
Novel plant genotypes	*Describe the methods by which all novel plant genotypes were produced. This includes those generated by transgenic approaches, gene editing, chemical/radiation-based mutagenesis and hybridization. For transgenic lines, describe the transformation method, the number of independent lines analyzed and the generation upon which experiments were performed. For gene-edited lines, describe the editor used, the endogenous sequence targeted for editing, the targeting guide RNA sequence (if applicable) and how the editor was applied.*
Authentication	*Describe any authentication procedures for each seed stock used or novel genotype generated. Describe any experiments used to assess the effect of a mutation and, where applicable, how potential secondary effects (e.g. second site T-DNA insertions, mosiacism, off-target gene editing) were examined.*

Magnetic resonance imaging

Experimental design

Design type	Structural MRI and resting-state functional MRI were used in this study.
Design specifications	See below.
Behavioral performance measures	No Behavioral data used in this study

Acquisition

Imaging type(s)	Functional, structural
Field strength	3 Tesla
Sequence & imaging parameters	For the HCP dataset, images were acquired on a customized 3T Siemens Skyra scanner. Resting-state fMRI runs were acquired in 2 mm isotropic voxel using multiband echo planar imaging (EPI), where each run endures 14 min and 33 s (repetition time (TR) = 720 ms, echo time (TE) = 33.1 ms). T1w and T2w images were all acquired at 0.7 mm isotropic resolution (T1w: TR = 2400 ms, TE = 2.14 ms, inversion time (TI) = 1,000 ms, flip angle (FA) = 8°; T2w: TR = 3200 ms, TE = 565 ms).<br>For the ABCD dataset, we leveraged ABCD-BIDS Community Collection release. Images in the discovery set were acquired across 21 sites with 3T scanners from three manufacturers namely Siemens, GE and Phillips in the test set. In

contrast, images in the validation set were acquired from only 3 sites with Siemens Prisma 3T MR scanner. Resting-state fMRI runs were acquired in 2.4 mm isotropic voxel using multiband EPI with slice acceleration factor 6 and resampled to 2 mm isotropic voxel. Each run lasted for 5 min (TR = 800 ms, TE = 30 ms). T1w and T2w images were all acquired at 1.0 mm isotropic resolution (T1w: TR = 2500 ms (Siemens and GE) / 6.31 ms (Philips), TE = 2.88 ms (Siemens) / 2.9 ms (Philips) / 2 ms (GE), TI = 1,060 ms, FA = 8°; T2w: TR = 3200 ms (Siemens and GE) / 2500 ms (Philips), TE = 565 ms (Siemens) / 251.6 ms (Philips) / 60 ms (GE)).

Area of acquisition: Whole brain scan was used.

Diffusion MRI: ☐ Used ☒ Not used

Preprocessing

Preprocessing software: The preprocessing of the HCP dataset used “minimal preprocessing pipelines" provided by HCP and the preprocessing of the ABCD dataset used modified HCP pipelines (https://zenodo.org/record/2587210).

Normalization: For vertex-wise analyses, surfaces were nonlinearly registered surface atlas (fs_LR) based on cortical folding patterns nonlinear algorithm. For voxel-wise analyses, T1w images were registered to atlas using affine and nonlinear registration with FSL's flirt and fnirt, respectively.

Normalization template: The template for surface-based registration was standard fs_LR_32k surface spherical atlas. The template for voxel-wise registration was FSL's T1_2_MNI152_2mm.

Noise and artifact removal: For the HCP dataset, T1w and T2w images were pre-processed under distortion correction, denoising, N4 bias correction, and MNI standard space registration. We leveraged skull-stripped T1w and T2w images for analysis. For resting-state fMRI, surface-based preprocessing of resting-state fMRI data included nuisance regression, temporal censoring, and spatial smoothing.
For the ABCD dataset, the additional preprocessing involves applying a general linear model to denoise the fMRI data, and enhancing the estimation of framewise displacement by the respiratory motion filter in the DBP. Additional details of the preprocessing procedures taken and pipeline used are publicly available (https://zenodo.org/record/2587210).

Volume censoring: For the ABCD dataset, participants containing more than 50% of frames with FD > 0.3mm were removed.

Statistical modeling & inference

Model type and settings: Univariate and multivariate analyses were carried-out in this study.

Effect(s) tested: Pearson's r for correlation analysis.

Specify type of analysis: ☒ Whole brain ☐ ROI-based ☐ Both

Statistic type for inference (See Eklund et al. 2016): Two-sample t-test, ANOVA and Pearson correlation were used in this study.

Correction: False discovery rate correction was employed in this study.

Models & analysis

n/a	Involved in the study
☐	☒ Functional and/or effective connectivity
☒	☐ Graph analysis
☐	☒ Multivariate modeling or predictive analysis

Functional and/or effective connectivity: Pearson correlation and Fisher r-to-z transformation was used for calculating functional connectivity.

Multivariate modeling and predictive analysis: SVM model was used for classification analysis.
